# Gender differences in active travel in major cities across the world

**DOI:** 10.1007/s11116-021-10259-4

**Published:** 2022-01-03

**Authors:** Rahul Goel, Oyinlola Oyebode, Louise Foley, Lambed Tatah, Christopher Millett, James Woodcock

**Affiliations:** 1 Transportation Research and Injury Prevention Centre, Indian Institute of Technology Delhi, New Delhi, India; 2 MRC Epidemiology Unit, University of Cambridge, Cambridge, UK; 3 Warwick Medical School, University of Warwick, Warwick, UK; 4 School of Public Health, Imperial College London, London, UK

**Keywords:** Active travel, Physical activity, Walking, Cycling, Public transportation, Gender, Age

## Abstract

There is lack of literature on international comparison of gender differences in the use of active travel modes. We used population-representative travel surveys for 19 major cities across 13 countries and 6 continents, representing a mix of cites from low-and-middle income (n = 8) and high-income countries (n = 11). In all the cities, females are more likely than males to walk and, in most cities, more likely to use public transport. This relationship reverses in cycling, with females often less likely users than males. In high cycling cities, both genders are equally likely to cycle. Active travel to access public transport contributes 30–50% of total active travel time. The gender differences in active travel metrics are age dependent. Among children (< 16 years), these metrics are often equal for girls and boys, while gender disparity increases with age. On average, active travel enables one in every four people in the population to achieve at least 30 min of physical activity in a day, though there is large variation across the cities. In general, females are more likely to achieve this level than males. The results highlight the importance of a gendered approach towards active transport policies. Such an approach necessitates reducing road traffic danger and male violence, as well as overcoming social norms that restrict women from cycling.

## Introduction

Walking and cycling are physical activities that can be incorporated in daily lives. People who engage in active travel tend to be more physically active (Roth et al. 2012; Sahlqvist et al. 2012). Physical activity reduces all-cause mortality and multiple chronic disease outcomes such as cardiovascular diseases, diabetes, and cancers (Bull et al. 2020; Pedersen and Saltin 2015; Warburton and Bredin 2017). Thus, widespread use of active travel across the population is beneficial for public health.

Walking and cycling can be for the whole journey or to access public transport. Studies have reported that use of public transportation is a significant contributor to physical activity (Rissel et al. 2012). A significant proportion of public transportation users chieve 30-min-a-day of physical activity (Besser and Dannenberg 2005; Patterson et al. 2019). They are also many times more likely to achieve 10,000 steps in a day compared to users of private motorised modes (Villanueva et al. 2008; Wener and Evans 2007). The level of activity may also vary by the type of public transportation. For example, train use leads to a much higher level of activity than the bus (Morency et al. 2011).

Daily mobility in a population is highly gendered (Alessandretti et al. 2020; Law 2002; Peters 2011). Average travel behaviour of women differs on from men in locations visited, trip purpose, trip distance, and mode of transport (Alessandretti et al. 2020; Gauvin et al. 2020; Goel et al. 2021; Law 2002; Ravensbergen et al. 2019). These differences result from space–time constraints, such as childcare and household tasks that fall disproportionately on women, that are also related to differences in employment levels (Kwan 2000; Peters 2011). Women are also more sensitive to traffic risk resulting in gender differences in the use of active travel (Aldred et al. 2017; Garrard et al. 2008a). Further, there are gender norms, cultural barriers, and fear of sexual harassment and assault by men that deter women from using certain modes of transport or travel at certain times of day (Iqbal et al. 2020; Phadke 2013). It is often while walking or cycling, or using public transport, when women feel most vulnerable during travel (Gekoski et al. 2017), thus making active travel for women a potential source of anxiety.

Given that gender inequalities vary across countries, it is likely that the gender gap, in both mobility generally and active travel specifically, varies greatly across the world. Althoff et al. (2020) used smartphone accelerometery data for ~ 100 countries and found large variation in gender inequality in walking (measured as daily number of steps). The highest gender inequality was reported for Qatar, Saudi Arabia, and Malaysia and the lowest in Sweden, Ukraine, and Russia. The study also concluded that population-wide activity inequality could be greatly reduced through increases in female activity alone. The study by Althoff and other such large-scale global studies, though useful to highlight gender gaps and its variation across the globe, often lack details such as the modes of transport that individuals use to attain their activity levels. There are very few studies that compared travel activity levels across a large range of countries especially a mix of low-and-middle and high-income countries. In this study we aim to address this gap in literature. Using travel surveys, we present active travel patterns for 19 cities across 13 countries located across 6 continents. We aim to answer the following research questions:

1. What are the gender differences across the cities in terms of use of active modes of transport for all trips and for trips to work?
2. What are the gender differences in active travel time and relative contributions of modes of transport to active travel time?
3. What are the gender differences in the proportion of the population achieving 30 min of physical activity from active travel?

The following includes the section on method that provide details of the datasets used, as well as the analysis conducted. This is followed by a section on results. Next, we present discussion and conclusions of the paper.

## Method

To answer these research questions, we used population-representative household travel surveys for 19 cities from 13 different countries in five different continents (Table 1). An exception is Accra in Ghana, where the dataset used is a time-use activity survey from which travel-related activities have been extracted as trips. We selected the settings based on data availability and to ensure representation of different regions of the world. The included countries represent a wide range of income levels. For example, India and Kenya had a per-capita income of less than US $2000 in 2015, Brazil and South Africa between US $6000 and US $10,000, and on the other extreme, Switzerland and the USA, higher than US $60,000. Among the countries listed, seven are low-and-middle income and the rest are high income. The latter include Australia, Chile, USA, and all three European countries. The details of travel surveys are presented in the Supplementary Material.

Travel survey datasets include demographic and socio-economic details of the individuals among the sampled households, and their travel diary on a given day, consisting of trips and corresponding stages. Within a trip, a stage is completed when a transfer is made from one mode to another. For example, a bus trip usually consists of three stages, with first stage of walking to bus stop, second stage of travelling by bus, and third stage of walking from bus stop to destination. Travel surveys often assign a date to the sampled households for which they report their travel activities in a diary format. In some surveys that involve face-to-face interviews of the respondents, respondents are asked to recall travel activities of the previous day. As a result, in travel surveys, there are individuals who report no travel activity, as is possible for a variety of reasons. This day for which respondents report their travel activities is referred to as ‘travel day’.

For this analysis, our focus is the use of active travel modes i.e. walking and cycling, and their contribution to population-level activity levels. Active modes of transport can be used to travel the whole trip, or one or more stages of a trip, for example, walking to public transport access points or to the parking lot. The ideal travel survey dataset for our analysis is when mode of transport and travel time are reported for each stage within a trip. Among the 19 cities, the datasets differ in reporting this detail. For some cities, all the required information is reported for the stages (Chicago, Delhi, London, Los Angeles, Melbourne, Mexico City, New York City, Zurich). For some, duration of total walking, done for part(s) of the trips, is reported for each trip (Sao Paulo and Bogota). In Buenos Aires, mode of travel for each of the stages is known, but duration of those stages is not. For some, stage-level data is not reported for any trip (Accra, Cape Town, Kisumu, Berlin, Hamburg, Munich, Cologne). In total there are 10 cities with data on stage-level active travel and 9 without it (see Table 1). We highlight these differences while presenting the results.

As many trips are multimodal in nature, ‘main mode’ of a trip is typically defined as the one with which a stage with the longest duration has been travelled. Some datasets reported main mode of transport for all trips. For the cities where it was not reported, we used the stage-level duration data to identify main mode. For cities, where stage-level data was reported, but no travel time (e.g. Buenos Aires), we used a hierarchy^1^ of travel modes that is often used for identifying main modes. For example, in a trip involving car and train, the latter is assumed as the main mode, and the car as an access mode. We defined work trips as those for which trip purpose or destination type was reported as work or work-related. For some datasets, the subsequent trip returning home was already classified as a work trip. For datasets, where these were not, we classified them as work trips. Given the diversity of cities represented in our dataset, we have a large range of modes of transport. We classified all the public transport modes into three categories— bus, metro, and train. We refer to shared taxis, trams, and dial-a-ride/para-transit as bus, as all these are road-based public transport modes. Metro includes metro trains and subways. Cycle rickshaws were categorised as ‘other’. We present results for three modes, two active travel modes— walk and bike, and one which usually involves some active travel time— public transport.

Following the research questions (RQ) mentioned above, we present five metrics of active travel use. These include mode share of all trips and for work trips (RQ 1), level of immobility (RQ 2) and active travel time per capita (RQ 2), percentage contribution of active travel time by main modes (RQ 2), and percentage of individuals achieving 30 min or more of daily active travel time (RQ 3). Mode share is calculated as the percentage share of trips by the main mode of travel. Level of immobility is the percentage of individuals who reported not going out of home on travel day, which could result from sickness, disability, voluntary decision to stay at home, or underreporting by survey respondents (Madre et al. 2007). Active travel time per capita is the total walking and cycling duration across all stages/trips divided by the total number of sampled individuals, including those who reported no travel. We classified population-wide active travel time into respective main mode categories, and express this as percentage share of total active travel time contributed by these modes. We present all results stratified by gender and four age groups—children (0–15 years), working age (16–60 years), older adults (> 60 years), and all age combined. Only for reporting mode share of trips to work, we do not classify results by age groups.

Among the metrics described above, active travel time per capita, percentage share of active travel time contributed by the main modes, and percent individuals attaining ≥ 30 min of active travel time on their travel day suffer from inconsistency of stagelevel data, which presents a challenge in comparing these metrics across the settings. For cities where stage-level data is not reported, this leads to underestimation of active travel that individuals engage in as part of public transport trips. Therefore, we developed the following method to harmonise these metrics across the cities. We calculated gender-specific trip-level average active travel time for all main modes (except walking and cycling) for each of the 10 cities where stage-level data is available. The averages were calculated for the following modes—bus, metro, train, taxi, car, and motorcycle. In some cities, one or more of these modes are not available. We calculated the median of those averages from 10 cities (Supplementary Material). Next, we multiplied these median values by the total number of trips of the respective main modes, for the set of cities where this data is not available. We refer to estimates derived using this calculation as ‘harmonised’ and for the 10 cities as ‘reported’. Using this method, we can harmonise the metrics of active travel time per capita and percentage share of active travel time, but not the metric of percent individuals attaining ≥ 30 min of active travel, as the latter needs harmonisation at the individual level. For this third metric, we only present data for the 10 cities. For sensitivity analysis, we used another approach in which cities were divided into three homogenous groups based on their income levels. Next, we assigned stage-level active travel time to cities from the average of the cities in their corresponding group. Within group 1, we used Bogota, Mexico City and Sao Paulo to approximate stage-level active travel time for Buenos Aires, Cape Town, and Santiago. In group 2, we used Delhi for Accra and Kisumu. In group 3 we used London and Zurich for the four German cities. For all calculations, we used survey weights reported in the datasets for representative estimates.

## Results

### Level of immobility by gender and age

**Fig. 1:**
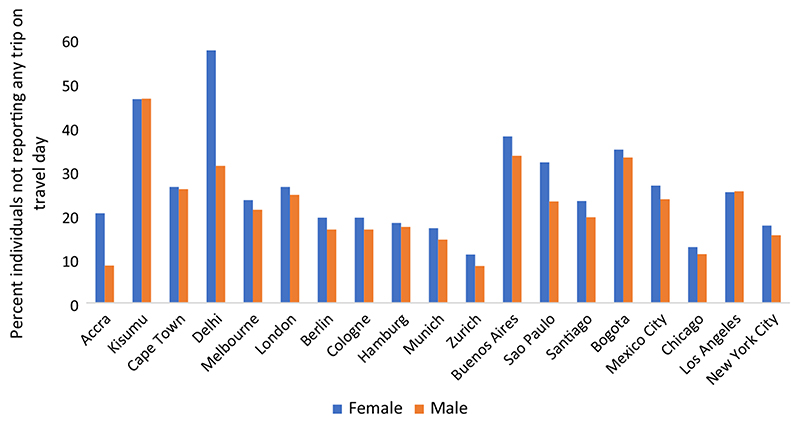
Level of immobility by gender for all age groups combined

Figure 1 presents the level of immobility for all age groups combined. The results for age-stratified analysis for this metric and all others are presented in Supplementary Material. In all cities, level of immobility is greater among females than males, with exceptions of Kisumu and Los Angeles, where the two are equal. On average, females have 4 percentage points higher level of immobility than males (26% and 22%, respectively). This difference is greatest in Delhi (26 percentage points), followed by Accra (12 percentage points) and Sao Paulo (9 percentage points). The gender difference in immobility varies across the age groups. The average divergence between the two groups is the highest among the older adults (8 percentage points higher for women), followed by working age group (5 percentage points higher for women), while among the children, girls have slightly lower level of immobility (less than one percentage point) than boys.

### Mode shares for all trips

In Table 2we present percentage share of all trips with walking, cycling, and public transport as main modes for all age groups combined. Note that the three proportions do not sum to 100 as there are also other modes of transport, for which the results are presented in Supplementary Material. Of the three modes of transport presented, walking is most common across the cities, followed closely by public transport. In comparison, cycling is used for a small minority of trips within most cities. There are, however, significant variations in mode share across the cities. Highest levels of walking are in Accra, Delhi, and Kisumu, with an average of 59% among females and 45% among males, and the lowest levels are in Chicago, Los Angeles, and Melbourne (average 14% and 13% for females and males, respectively). In all the cities, females have higher likelihood to walk and, on an average, this likelihood is 23% greater than that of men (calculated as the average of the ratios of the mode share of females to males). Among the three age groups, children have the highest levels of walking, and working age group and older adults have slightly lower but similar levels of walking. Gender difference in walking is the lowest among children, highest among working age group, and slightly lower than working age group among the older adults.

Among the three modes of transport, levels of cycling show greatest variation across the cities. The lowest levels of cycling are in Accra and Cape Town, with almost no cycling among females and less than a percent among males. The highest levels are in the four German cities with an average of 14–15% for the two gender groups. The gender differences are the highest for this mode of transport, and in a direction opposite to that of walking. On average, females are half as likely as males to cycle. The notable exceptions are the German cities of Berlin, Munich, and Hamburg, where females are **more** likely to cycle than males. Cycling levels are generally the highest among the working age group followed by children, and the lowest among older adults. The gender differences vary greatly over the age groups. Among children, girls are 30% less likely to cycle than boys. Among working age groups and older adults, gender differences are equally high, with about 50% lower likelihood among women than men.

Highest levels of public transportation mode shares are in the Latin American cities of Bogota, Buenos Aires, Mexico City, Santiago, and Sao Paulo, with an average of 39% among females, and 37% among males. These levels are many times greater than in Chicago, Los Angeles, and Melbourne, which have the lowest levels of public transport use (average of 7% for the two gender groups). In all the cities, except Delhi and Mexico City, females are more likely to use public transport. On an average, likelihood to use public transport among females is about 6% higher than among males. The use of public transport is lowest among the children, highest among the working age, and slightly lower among the older adults than the working age. Gender difference in the use of public transportation is highest among older adults. In this age group, women are, on average, 26% more likely to use public transportation than men. Among children and working age group, females are only 5–6% more likely, on average.

### Mode shares of work trips

In Table 3, we present modes shares of the walking, cycling and public transport for work trips. For this analysis, we present results only for all age groups combined, as work trips exclude children and most older adults. Among work trips, public transportation is the most common mode of transport, walking is the second most common, followed by cycling. Women have on average 25% higher likelihood to walk to work than men—similar to the finding for all trips (Table 2). Highest levels of walking are in Accra, Delhi, and Kisumu with an average of 41% among women and 28% among men. Lowest levels of walking to work are in Chicago, Hamburg, Los Angeles, and Munich, with an average of 6–7% among women and men.

The levels of public transportation among women are the highest in Bogota, Buenos Aires, Mexico City, New York City, and Santiago, ranging from 57 to 68%, while among men, these levels range from 37 to 54% in those cities. In contrast, in Chicago and Los Angeles, use of public transportation among women is 8–12%, and among men, less than 10%. Women are on average 21% more likely than men to use public transport to travel to work, and this gender gap is much higher than in all trips combined. The gender differences in the use of public transport are the highest in the Latin American cities.

Similar to the finding in the previous section, the four German cities have the highest levels of cycling to work, and these levels are even higher than their corresponding values in all trips combined, presented in Table 3. Bogota and Delhi are the other two cities with high levels (10–12%), though only among men. In general, likelihood of cycling to work is higher than the likelihood of cycling in general. On average, women are half as likely as men to cycle to work, which is the same level of gender gap as we found among all trips.

### Active travel time

**Fig. 2:**
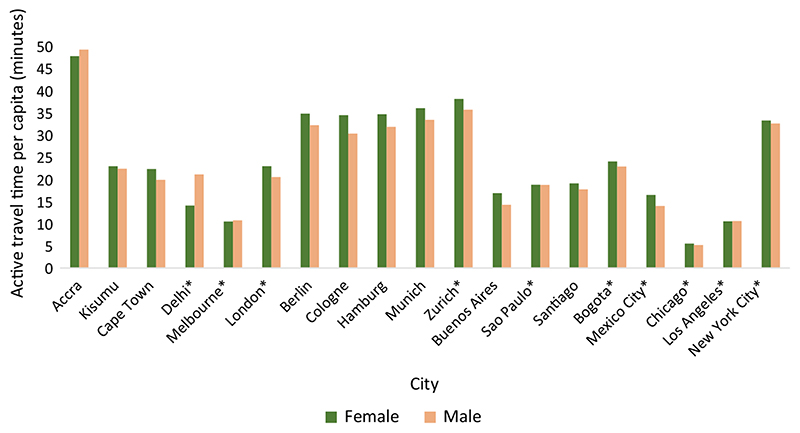
Active travel time per capita by gender for all age groups combined (asterisk represent those cities which use reported data, and others represent harmonised estimates)

Figure 2 presents harmonised active travel time per capita. The cities indicating ‘reported’ used reported stage-level travel time and those indicating ‘harmonised’ used the estimated travel time for stages (see Method section). In all cities except Accra and Delhi, females spend more time travelling actively than males, though the gender differences in most cities are small. On average, females have 5% higher active travel time per capita than males, with values of 24.4 min and 23.3 min, respectively. Chicago has the lowest levels with 5.5 min among females (f) and 5.2 min among males (m), followed by Melbourne (10.5: f; 10.7: m) and Los Angeles (10.5: f, 10.6: m). The highest levels are in Accra (48: f; 50: m) and in Zurich (38: f; 36: m). The gender difference is the highest in Delhi and Buenos Aires, though in opposite directions. In the former, females have 33% **lower** levels of activity than males, and in the latter, females have 15% **higher** levels of activity than males.

Among the age groups, working age adults have the highest level of active travel time per capita, and females have a higher level than males (26.1 min and 24.3 min, respectively). Older women have the lowest levels among all gender and age sub-groups, and the gender difference within this age group is also the highest— women have on average 16% **lower** active travel time per capita than males. This gender difference among older adults is in an opposite direction to the other two age groups— among children and working age groups, females have **higher** levels of activity than males. In Delhi and Buenos Aires, which have the highest gender difference for all age groups combined, activity levels are equal among children. It is in the higher age groups that the gender gap widens for the two cities, except in Buenos Aires, two gender groups are again equal among older adults. Using the alternate method for harmonisation, we found that the average results of most cities are within 4 percent of the estimates obtained using the main approach (see SI). Thus, our results are not sensitive to the assumption used for harmonisation.

In Table 4, we present share of total active travel time (walking and cycling) contributed by the three categories of main mode—walk, cycle, and public transport. Note that the percentages across the three modes do not sum to 100% as some part of active travel (2–3%) is contributed by other modes of transport, for example, cars and motorised two-wheelers. On average, females obtain 62% of their active travel time from those trips in which they walked all the way. In comparison, this share is 54% among males. The three cities with the highest contribution from walking among females are Delhi (91%), Accra (81%), and Kisumu (76%). Among males, highest contribution from walking is in Accra (80%), Kisumu (70%), and Zurich (71%).

Cycling contributes an average of 8% of total active travel time among females across the cities, while it contributes about twice as much (15%) among males. The four German cities have the highest contribution of cycling to active travel time, with an average of 24% among females and 29% among males. The average contribution of public transport to total active travel time is 28–29% for the two gender groups, and the gender gap is the lowest among the three modes of transport. The three cities with the highest contribution of public transport to active travel time for females are Buenos Aires, New York City and Santiago (43–51%), and for males are Buenos Aires, Mexico City and Santiago (46–55%). Delhi has the lowest contribution by public transport for females (8%) and Zurich for males (14%).

Among the three age groups, walking has the highest contribution to active travel time among children, and the lowest among working age group. Public transport and cycling have the highest contribution among the working age group. The lowest share of public transport is among children, and the lowest share of cycling is among older adults. The gender differences are the lowest among children for all the three modes. The highest gender gap for walking is among working age adults, and the highest gender gap for cycling is among working age and older adults. For these two age groups, the share of cycling to active travel time among females is half that of males.

### Individuals gaining 30 min or greater of active travel time

**Fig. 3:**
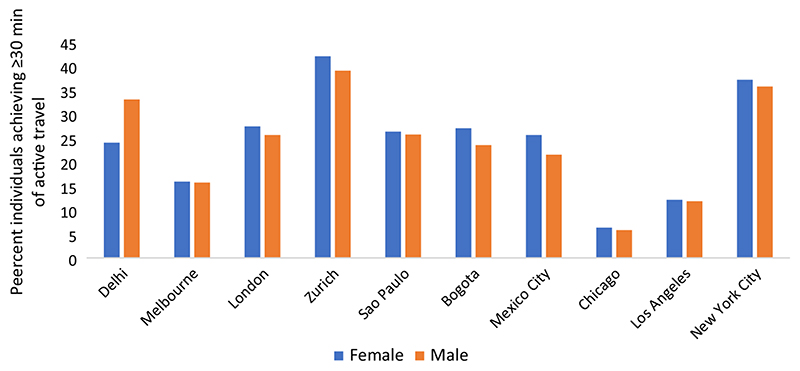
Percentage of individuals attaining at least 30 min of active travel time

In Fig. 3, we present the percentage of individuals across all age groups reporting at least 30 min of daily active travel time. We present this metric for the 10 cities for which stage-level duration of travel was available. Compared to the full set of 19 cities, these 10 cities include representation of all the regions except Africa. On an average, one in every four people achieve 30 min of daily active travel. The gender gap in achieving this level is the highest in Delhi where 24% females achieve 30 min of active travel compared to 33% males. Delhi is also the only city where this proportion is lower among females than among males. Excluding Delhi, on an average, females have 7% higher likelihood to achieve 30 min of active travel than males. Among these, the highest gender gap is in the Latin American cities of Bogota and Mexico City—27% females versus 24% males in the former, and 26% females and 22% males in the latter.

Among the three age groups, working age adults are most likely to achieve at least 30 min of active travel and older adults have the lowest, with children in the middle. Children are most gender equal among the three age groups. In Delhi, where overall gender gap is the highest, there is no gender gap among the children. In Mexico City and Bogota also, the gender gap among children is much smaller than the other age groups. Working age group has similar gender gap (females more likely) as all age groups combined, and among older adults, the gender gap is in an opposite direction, and females are 8% less likely than males to achieve 30 min of active travel.

## Discussions

### Summary of findings

In most cities, females have greater level of immobility than males. In some cities of low-and-middle income countries (Delhi, Accra, Sao Paulo), this gender gap is much wider. Females in all cities are more likely to walk for all trips combined and, in most cases, more likely to use public transport compared to males. This relationship reverses in cycling and females are always less likely users than males, except in German cities. For commuting to work, women’s use of public transportation is significantly greater than men. Per capita active travel time is highest in Accra and New York City, followed by the German cities. About one-third of total active travel time across the cities is from active travel related to public transport. In some of the Latin American cities, this proportion is as high as 50 percent. On average, daily travel results in one in every four people in the population to achieve at least 30 min of active travel, though there is large variation across the cities. In general, females are more likely to achieve this level than males. The gender differences of active travel metrics are often age dependent. Children (< 16 years) are often gender equal and gender disparity increases with age.

An interesting finding is that despite large gender differences in the use of modes of transport, as expressed by mode shares, overall levels of active travel time are remarkably similar. We also found regional differences in the sources of active travel. In Latin America, public transport is most dominant, in Germany, cycling, and in Africa and Asia, walking. German cities are clear outliers with their high levels of cycling as well as gender equality in its use. This is in line with a global comparison of cycling in the cities (Goel et al. 2021) that reported greater gender equality in cycling use in the cities with high levels of cycling. Cities in the US (except New York City) and Australia have exceptionally low use of all the three modes of transport. There is no indication that the use of any one mode explains the activity levels of population. For example, Latin American cities with greater contribution by public transport has similar levels of active travel time as Indian or African cities dominated by walking. Similarly, New York city dominated by public transport has similar levels of active travel time as German cities dominated by cycling.

### Meaning of our findings

Our study results highlight the levels of physical activity that can be achieved from daily travel as well as the modes of transport that contribute to this activity. While the former is a determinant of health, the latter determines the access to the city. In Bogota, for example, females and males have almost the same levels of active travel time (24 and 23 min per capita). However, males gain a much greater proportion of active travel time by cycling than females (28% and 7%), and therefore the former can achieve far greater spatial access. Similarly, in Delhi, females gain almost no active travel from cycling, while men gain up to 20% of all active travel time. Thus a focus on active travel time alone underestimates gender differences in travel behaviour and its impact on access. Gender inequality often results in lower access to vehicles for women, which increases their dependence on active travel and public transport (Peters 2011). Within active travel, lack of safe environments, cultural norms, and fear of harassment discourage women from cycling (Garrard et al. 2008a; Goel et al. 2021; Iqbal et al. 2020; Prati 2018; Ravensbergen et al. 2020). Therefore, in populations with high levels of gender inequality, low vehicle ownership and poor or no infrastructure for cycling, women are particularly disadvantaged. Gender inequality in cycling use and poor infrastructure are interrelated. Cycling without appropriate provision can be seen as out of place and stigmatised (Aldred 2013), therefore, women who already suffer disadvantage in society are likely to risk compounding their disadvantage by using a stigmatised mode.

In Delhi, for example, 66% of trips by females are by walking, compared to 40% by males, and almost all cycling trips are made by males (Goel et al. 2021). This lack of access to cycling and greater dependence on slower modes of transport, combined with women’s disproportionate share of household responsibilities, compounds gender inequalities (Anand and Tiwari 2006; Peters 2011). A contrasting example is Berlin, which is a much more gender equal society *and* has safe infrastructure for cycling. As a result, the city has high levels of gender equality in both active travel time as well as the modes of transport used. Similar results were reported by (Goel et al. 2021) for many settings (countries and cities) in Western Europe with high levels of gender equality and safe infrastructure of cycling. These regional differences across the world have also been highlighted by a global study analysing GPS traces of individuals (Alessandretti et al. 2020). The study found that Western European countries have the highest gender equality in daily mobility, expressed in terms of spatial coverage of movement, while countries such as Saudi Arabia, India, South Korea, and Chile are among the least equal. Thus, the efforts to improve active travel among the population should ensure a city that is accessible by walking, cycling and public transport, and this requires reducing road traffic danger and risk of harassment and assault by men. Car use can provide some protection from danger (though increasing risks to others), and gender equity in car access should be a goal. However, in lower income cities, car ownership is far out of the reach of the majority of the population, whilst in higher income cities car ownership and use should be reduced to tackle climate change and its other adverse effects. Reducing these sources of danger and challenging norms that stop women from cycling, can strive to achieve gender equity in activity levels and in access to the city.

By presenting active travel time as well as its contributors in terms of modes of transport, our study results also highlight a strong link between transport and public health. The results show that transportation system of a city is a strong determinant of the amount of active travel time gained by individuals, how they achieve this time, and how this translates to gender differences in access. The results add empirical evidence to the findings by (Salvo et al. 2021), in which authors highlighted the importance of transport infrastructure in the promotion of population-level physical activity in the cities, though their results were not gender stratified.

### Unanswered questions and future research

We found results of Delhi at odds with other cities in our analysis. In Delhi, the level of immobility of women is much lower than men. Also, only in Delhi, females had much lower levels of active travel activity than males. In all other cities, it was the reverse, and the gender gap was much smaller. One explanatory factor could be that India has one of the lowest levels of paid work-participation rate among women in the world (Deshpande and Kabeer 2019). According to the Census data of India, women constituted only 17 per cent of all workers in urban areas who reported travelling to work outside home (Census-India 2016). An analysis of time-use survey in Pakistan, a neighbouring South Asian country, also reported that immobility level of women was 55% compared to only 4% of men (Adeel et al. 2013). The same study reported men make twice as many daily trips as women. Reasons for high level of female immobility in South Asia needs further investigation in future research.

Travel surveys ask for travel-related activities in a diary format for a specific day, and only less frequently, for 2 days or a full week. At the population level, such surveys represent travel activity for a typical day. This is markedly different from standardised physical activity surveys that ask individuals about their physical activity behaviour for the past or a typical week. One day travel diary surveys may therefore underestimate active travel that individuals may engage in less frequently, such as walking to the park. This is one reason why physical activity prevalence from travel diaries and physical activity questionnaire may not be comparable. Additionally, there are quality issues in the survey datasets that may have some impact on the estimates of travel physical activity. For example, while some surveys were conducted across the year, many were conducted only during certain period of the year. This may bias our results if there is seasonality in the use of active travel.

As a result of single-day reporting in travel surveys, level of immobility (Fig. 1) has a significant effect on population-level outcomes such as average active travel time per capita (Fig. 2). If an individual does not report any travel activity on the assigned day, he/she is excluded from any active travel. The absence of travel activity could be because on the survey day the person could not go out of home or did not need to. It could also result from underreporting by respondents, for example, due to proxy responses (Madre et al. 2007; Wang et al. 2021). A large proportion of individuals not reporting any trip would bring down the population-level activity levels, even if those who did report a trip were predominantly engaging in active travel. Future studies should investigate the impact that duration of travel diary (1 day, 2 days, or a week) has on the estimates of active travel duration as well as resulting gender differences, and whether such methods of survey reduce underreporting. There is also a need to develop methods to harmonise estimates of travel diary with those reported by physical activity surveys.

## Conclusions

We used population-representative travel surveys for 19 major cities across 13 countries and 6 continents, representing a mix of cities from low-and-middle income (n = 8) and high-income countries (n = 11). We found that in many cities there are large gender differences in the modes of transport that individuals use to attain travel physical activity even if the levels of activity are almost the same. We argue that these gender differences stem in part from social norms that restrict women to certain modes of transport combined with lack of safe infrastructure for active travel, most notably cycling. Evaluations of transport policies in general, and active travel policies in particular, should focus on the pathways that result in reduced risk of traffic danger, harassment, and assault while using active travel modes and normalise cycling amongst women.

## Supplementary Material

The online version contains supplementary material available at https://doi.org/10.1007/s11116-021-10259-4.

Supplementary Material

## Figures and Tables

**Table 1:** List of cities included in the analysis ordered by the region

City	Country	Region	Stage-level data*
Accra	Ghana	Africa	No
Kisumu	Kenya	Africa	No
Cape Town	South Africa	Africa	No
Delhi	India	Asia	All information
Melbourne	Australia	Australia	All information
London	England	Europe	All information
Berlin	Germany	Europe	No
Cologne	Germany	Europe	No
Hamburg	Germany	Europe	No
Munich	Germany	Europe	No
Zurich	Switzerland	Europe	All information
Buenos Aires	Argentina	Latin America	No
Sao Paulo	Brazil	Latin America	Only walking duration
Santiago	Chile	Latin America	No
Bogota	Colombia	Latin America	Only walking duration
Mexico City	Mexico	Latin America	All information
Chicago	USA	North America	All information
Los Angeles	USA	North America	All information
New York City	USA	North America	All information

* Travel survey includes stages within a trip

**Table 2:** Mode shares for all trips by gender and for all age groups combined (colour coding for two gender groups combined and specific to a mode)

City	Country	Region	Walking (%)		Cycling (%)		Public Transport (%)	
			Female	Male	Female	Male	Female	Male
Accra	Ghana	Africa	60.6	56.4	0.1	0.8	28.0	23.6
Kisumu	Kenya	Africa	50.5	38.3	2.1	7.0	23.5	24.1
Cape Town	South Africa	Africa	30.2	29.2	0.1	0.4	32.8	27.3
Delhi	India	Asia	66.2	39.9	1.1	6.9	17.3	24.0
Melbourne	Australia	Australia	17.0	16.3	1.2	2.3	8.8	8.8
London	England	Europe	33.7	29.3	1.3	4.0	27.4	27.2
Berlin	Germany	Europe	26.8	24.1	15.0	13.2	23.7	19.7
Cologne	Germany	Europe	26.0	22.9	14.4	15.0	14.4	13.7
Hamburg	Germany	Europe	26.9	23.1	13.9	13.4	18.8	17.7
Munich	Germany	Europe	23.4	21.6	16.8	15.8	21.9	19.1
Zurich	Switzerland	Europe	37.1	32.1	5.7	6.9	17.7	14.5
Buenos Aires	Argentina	Latin America	32.1	22.3	2.2	4.3	47.7	41.7
Sao Paulo	Brazil	Latin America	34.8	28.1	0.2	1.1	38.2	32.5
Santiago	Chile	Latin America	33.9	24.0	2.5	5.1	34.1	31.1
Bogota	Colombia	Latin America	41.0	29.1	2.5	9.0	38.3	35.0
Mexico City	Mexico	Latin America	40.3	23.3	1.0	3.2	37.8	45.3
Chicago	USA	North America	11.1	10.3	0.5	1.3	6.3	6.9
Los Angeles	USA	North America	13.2	12.0	0.7	2.0	6.0	6.0
New York City	USA	North America	31.8	30.2	0.7	1.8	32.0	30.4

**Table 3:** Mode shares for work trips by gender and for all age groups combined

City	Country	Region	Walking (%)		Cycling (%)		Public Transport (%)	
			Female	Male	Female	Male	Female	Male
Accra	Ghana	Africa	47.3	30.5	0.3	1.1	36.3	39.2
Kisumu	Kenya	Africa	37.1	27.6	1.9	7.0	27.0	23.6
Cape Town	South Africa	Africa	15.3	15.6	0.4	0.8	41.7	31.5
Delhi	India	Asia	37.6	25.7	2.2	10.0	34.4	28.8
Melbourne	Australia	Australia	8.5	7.0	1.6	2.6	19.1	15.0
London	England	Europe	16.6	11.8	2.6	6.2	49.1	42.9
Berlin	Germany	Europe	6.5	7.5	19.0	16.3	40.5	33.7
Cologne	Germany	Europe	10.0	10.0	17.5	18.3	25.3	26.2
Hamburg	Germany	Europe	7.1	6.2	16.9	14.6	35.6	32.3
Munich	Germany	Europe	6.0	6.7	18.0	19.4	40.7	32.0
Zurich	Switzerland	Europe	23.1	19.3	8.1	8.9	33.5	24.3
Buenos Aires	Argentina	Latin America	13.6	9.3	2.2	5.6	67.9	49.5
Sao Paulo	Brazil	Latin America	23.7	18.2	0.2	1.6	50.9	36.7
Santiago	Chile	Latin America	14.3	10.2	2.4	6.1	56.8	42.6
Bogota	Colombia	Latin America	15.0	10.3	3.0	11.7	61.3	42.8
Mexico City	Mexico	Latin America	15.4	11.3	1.0	3.7	59.0	53.5
Chicago	USA	North America	8.5	6.4	0.6	1.2	11.6	8.9
Los Angeles	USA	North America	6.8	5.2	0.5	2.5	8.1	7.2
New York City	USA	North America	10.9	9.5	0.7	2.0	57.0	54.2

**Table 4:** Percentage share of total active travel time contributed by different main modes (harmonised to account for active travel while using PT; colour coding specific to gender for all modes combined)

City	Country	Region	Active travel for public transport	Females			Males		
				Walking	Cycling	Public Transport	Walking	Cycling	Public Transport
Accra	Ghana	Africa	harmonised	81.1	0.0	17.8	79.5	1.2	19.3
Kisumu	Kenya	Africa	harmonised	76.2	3.7	18.4	69.6	10.9	19.5
Cape Town	South Africa	Africa	harmonised	65.6	0.4	31.8	69.1	1.0	29.9
Delhi	India	Asia	reported	90.5	1.5	6.6	61.2	21.3	16.4
Melbourne	Australia	Australia	reported	60.4	6.9	31.2	53.4	14.3	30.4
London	England	Europe	reported	62.2	3.0	30.7	51.4	10.0	34.5
Berlin	Germany	Europe	harmonised	47.5	23.1	27.4	46.3	27.7	26.0
Cologne	Germany	Europe	harmonised	57.9	22.9	16.6	50.3	30.3	19.3
Hamburg	Germany	Europe	harmonised	52.8	22.3	22.6	47.6	27.8	24.6
Munich	Germany	Europe	harmonised	46.3	25.9	25.7	43.5	30.5	26.0
Zurich	Switzerland	Europe	reported	75.1	8.1	14.5	71.3	12.5	14.2
Buenos Aires	Argentina	Latin America	harmonised	44.0	2.7	51.3	36.9	8.3	54.7
Sao Paulo	Brazil	Latin America	reported	48.6	0.3	42.2	44.2	3.4	39.8
Santiago	Chile	Latin America	harmonised	47.4	3.9	46.1	39.8	13.9	46.3
Bogota	Colombia	Latin America	reported	67.2	6.5	23.4	49.1	28.0	19.2
Mexico City	Mexico	Latin America	reported	63.9	1.8	33.2	43.5	8.2	47.2
Chicago	USA	North America	reported	72.6	4.1	20.5	66.7	10.5	20.2
Los Angeles	USA	North America	reported	70.8	5.6	22.3	59.5	18.7	20.6
New York City	USA	North America	reported	51.0	1.7	42.9	48.9	3.6	41.9

## Footnotes

1 Train > Metro > Bus > Taxi > Car > Motorcycle > bicycle > walking.
