# Supplementary material for: Gender differences in active travel in major cities across the world
Source: Transportation (Amst). Author manuscript; Available in PMC 2023 Apr 7. (PMC7614415; doi:10.1007/s11116-021-10259-4)
Supplement: Supplementary Material [file EMS141041-supplement-Supplementary_Material.docx]

**Table A1: Sources of travel surveys (cities in alphabetical order)**

| **Country/City** | **Sampling and months of survey** | **Included in study** | **Survey Name and Access** | **Sample Unit** | **Response Rate** | **Survey method** | **Duration of Travel Diary** | **Survey Frequency** | **Years Analysed** | **City population** | **Sample Size (number of individuals)** |
| --- | --- | --- | --- | --- | --- | --- | --- | --- | --- | --- | --- |
| **Accra, Ghana** | Stratified random sampling | Greater Accra Metropolitan area | Ghana Time use survey, Ghana Statistical Service | Household in Ghana | 80% | face to face | 1 random day | One time | 2009 | 2,967,315 | 910 |
| **Berlin, Germany** | Random digit dialling; Survey from May to September | City | The MiD (Mobilität in Deutschland) survey; https://daten.clearingstelle-verkehr.de/order-form.html. | Households in Germany | 20% (2008) | CATI | 1 nominated day | Quinquennial | 2017 | 3,520,031 | 4099 |
| **Bogota, Colombia** | Stratified random sampling; February to August | All | Mobility Survey - Prudecia Bogota; https://www.simur.gov.co/portal-simur/datos-del-sector/encuestas-de-movilidad/ | Households in Bogota |  | Self | 1 random day | Quinquennial | 2015 | 7,400,000 | 52173 |
| **Buenos Aires, Argentina** | Probabilistic, multi-stage and stratified sample | All | Household Mobility Survey 2009-2010: Mobility in the Buenos Aires Metropolitan Area; http://datar.info/pt_BR/dataset/enmodo-2014 | Households in Greater Buenos Aires |  | Face to face | 1 random day | One time | 2009-2010 | 12,985,885 | 70321 |
| **Cape Town, South Africa** | Stratified, systematic random sampling, March to June | City | National Household Travel Survey; www.statssa.gov.za | Households in Cape Town | 89% | Face to face | 1 random day | Quinquennial | 2013 | 3,740,000 | 7144 |
| **Chicago, USA** | Random Digit Dialling and Directory/Address- based sample; January to March | Chicago city: Cook and Dupage counties | Chicago Regional Household Travel Inventory; https://www.cmap.illinois.gov/data/transportation/travel-survey | Households within the 11-counties: Chicago and NW Indiana | 30% | CATI | 1 or 2 days | One time | 2007 - 2008 | 2,696,000 | 32343 |
| **Cologne, Germany** | Same as for Berlin | City | Same as for Berlin |  |  |  |  |  |  | 1,005,775 | 6224 |
| **Delhi, India** | Two-stage stratified random sampling; March to September | City | accessed through Transportation Research and Injury Prevention Programme (TRIPP) at Indian Institute of Technology Delhi | Households in Delhi | 65% | Face to face | 1 random day | One time | 2013 | 16,700,000 | 6684 |
| **Hamburg, Germany** | Same as for Berlin | City | Same as for Berlin |  |  |  |  |  |  | 1,787,408 | 17008 |
| **Kisumu, Kenya** |  | City | Kisumu Household travel Survey; Institute for Transportation & Development Policy | Households in Kisumu |  | Face to face | 1 random day | One time | 2016 | 560,000 | 5767 |
| **London, England** | Stratified random sampling using Postcode Address File; conducted throughout the year | All | London Travel Demand Survey; [ltdsenquiries@tfl.gov.uk](mailto:ltdsenquiries@tfl.gov.uk) https://www.kisumu.go.ke/wp-content/uploads/2020/12/Kisumu-Sustainable-Mobility-Plan-200716.pdf | Households in London | 50% | Face to face | 1 nominated day | Continuous rolling survey | 2011-2013 | 8,200,000 | 57677 |
| **Los Angeles, USA** | Random sampling from addresses; throughout the year | Los Angeles County | 2010–2012 California Household Travel Survey; https://www.nrel.gov/transportation/secure-transportation-data/tsdc-california-travel-survey.html | Households in California State | 4.9% | CATI, online, or self | 1 nominated day | one time | 2012 | 3,792,621 | 21115 |
| **Melbourne, Australia** | Stratified cluster design; July to July | Metropolitan Melbourne | The Victorian Integrated Survey of Travel & Activity (VISTA); https://transport.vic.gov.au/about/data-and-research/vista | Households in 5 Metropolitan Melbourne regions and the Regional City region | 46.3% | Self | 1 nominated day | 2015-16 is final phase of VISTA project that started in 2012 | 2012-13 to 2017-18 | 2,300,000 | 61321 |
| **Mexico City, Mexico** | Stratified cluster design; January to March | Metropolitan area of ​​the Valley of Mexico | Encuesta Origen-Destino en Hogares de la Zona Metropolitana del Valle de México 2017; https://www.inegi.org.mx/programas/eod/2017/ | Households in the Mexico City Metropolitan Area | 83% | Face to face | 1 random day | One time | 2017 | 19,383,068 | 200117 |
| **Munich, Germany** | Same as for Berlin | City | Same as for Berlin |  |  |  |  |  |  | 1,450,381 | 17180 |
| **New York city, USA** | Random address sampling; September to November | New York City | 2010/2011 Regional Household Travel Survey; Selected New York City residents for the analysis; http://www.njtpa.org/Data-Maps/Modeling-Surveys/Household-Travel-Survey/Data-Disclaimer.aspx?ext=. | Households in 28 counties in New York / New Jersey / Connecticut metropolitan area |  | Self | 1 - nominated day | One time | 2010 -2011 | 8,175,000 | 11707 |
| **Santiago, Chile** | Probability Proportional to Size; July 2012 to Nov 2013 | All | Encuesta Origen Destino de Viages 2012; http://www.sectra.gob.cl/encuestas_movilidad/encuestas_movilidad.htm#Results%20in%20reports%20and%20microdata%20available.High-income%20country | Households in Gran Santiago |  | Self | 1 nominated day | Decade | 2012 | 665,1735 | 60054 |
| **Sao Paulo, Brazil** | Stratified random sampling | City | São Paulo Metropolitan Region Mobility Survey; https://www.mobilize.org.br/estudos/137/pesquisa-de-mobilidade-da-regiao-metropolitana-de-sao-paulo-2012.html | Households in Sao Paulo Metropolitan area |  | Face to face | 1 random day | Decade | 2012 - 2013 | 20,012,000 | 24534 |
| **Zurich, Switzerland** | Random selection using telephone; throughout the year | Zurich agglomeration (2012) | Microcensus Mobility and Transport 2015; Office fédéral de la statistique OFS, Section Mobilité | Households in Switzerland | 53% | CATI | 1 nominated day | Quinquennial | 2015 | 1,660,000 | 5453 |

**Table A2: Active travel time (walking+cycling) per trip in minutes**

|  | **Walking** | | **Cycling** | | **Bus** | | **Metro** | | **Train** | | **Taxi** | | | **Car** | | | **Motorcycle** | |
| --- | --- | --- | --- | --- | --- | --- | --- | --- | --- | --- | --- | --- | --- | --- | --- | --- | --- | --- |
| **City** | **Female** | **Male** | **Female** | **Male** | **Female** | **Male** | **Female** | **Male** | **Female** | **Male** | **Female** | **Male** | **Female** | | **Male** | **Female** | | **Male** |
| Accra | 23.8 | 19.3 |  | 20.4 |  |  |  |  |  |  |  |  |  | |  |  | |  |
| Kisumu | 21.8 | 25 | 25.2 | 21.6 |  |  |  |  |  |  |  |  |  | |  |  | |  |
| Cape Town | 26.9 | 26.2 | 33.7 | 25 |  |  |  |  |  |  |  |  |  | |  |  | |  |
| Delhi | 20.6 | 21.9 | 20.7 | 44 | 12.7 | 15.3 | 21.4 | 20.4 |  |  | 3.4 | 4.4 |  | |  |  | |  |
| Melbourne | 13.4 | 13.2 | 22.2 | 24.9 | 12.7 | 12.5 |  |  | 15.2 | 15.7 |  |  | 0.1 | | 0.1 |  | |  |
| London | 17.3 | 15.5 | 21.9 | 22 | 8.5 | 8.7 | 12.3 | 12.4 | 14.7 | 14.7 | 1.5 | 1.1 | 1 | | 0.9 | 1.1 | | 0.5 |
| Berlin | 21.3 | 20.6 | 18.4 | 22.6 |  |  |  |  |  |  |  |  |  | |  |  | |  |
| Cologne | 26.5 | 21.6 | 19 | 19.8 |  |  |  |  |  |  |  |  |  | |  |  | |  |
| Hamburg | 23.2 | 21.1 | 19 | 21.3 |  |  |  |  |  |  |  |  |  | |  |  | |  |
| Munich | 24.1 | 21.4 | 18.7 | 20.5 |  |  |  |  |  |  |  |  |  | |  |  | |  |
| Zurich | 23.1 | 22.5 | 16.1 | 18.4 | 7.9 | 8 |  |  | 11.6 | 12.1 |  |  | 0.6 | | 0.4 |  | |  |
| Buenos Aires | 15.2 | 15.4 | 14.2 | 18.1 |  |  |  |  |  |  |  |  |  | |  |  | |  |
| Sao Paulo | 14.9 | 14.9 | 22.2 | 30.6 | 10.8 | 10.3 | 13.7 | 13.9 | 16 | 15.3 | 3.1 | 3.1 | 3.5 | | 3.2 | 2.9 | | 2.8 |
| Santiago | 13.3 | 14.5 | 15.2 | 23.8 |  |  |  |  |  |  |  |  |  | |  |  | |  |
| Bogota | 23.8 | 23.5 | 37.1 | 43.1 | 8.8 | 7.6 |  |  |  |  | 3.2 | 2.4 | 1.8 | | 1.8 | 1.8 | | 1.7 |
| Mexico City | 15.4 | 16.2 | 16.8 | 22.5 | 11.8 | 12.1 | 13.3 | 13.6 | 13.9 | 14.7 | 1.3 | 1.3 | 0.2 | | 0.2 | 0.8 | | 0.3 |
| Chicago | 14.6 | 14.9 | 19.6 | 19.1 | 9.7 | 9.7 |  |  | 10.7 | 11.3 |  |  |  | |  |  | |  |
| Los Angeles | 19.7 | 19.3 | 27.8 | 37 | 15.6 | 15.5 | 17.4 | 18.1 | 17 | 18.4 |  |  | 0.1 | |  |  | | 0.4 |
| New York City | 18.7 | 18.6 | 26.9 | 23.1 | 14.7 | 16.7 | 17.5 | 17.2 | 15.2 | 21.5 | 3.2 | 3.8 | 1.3 | | 1.6 |  | |  |

**Table A3: Level of immobility (percentage individuals reporting making no trip on travel day) by gender and age**

| **City** | **Country** | **Region** | **All age groups combined** | | **Children** | | **Working age group** | | **Older adults** | |
| --- | --- | --- | --- | --- | --- | --- | --- | --- | --- | --- |
|  |  |  | **Female** | **Male** | **Female** | **Male** | **Female** | **Male** | **Female** | **Male** |
| Accra | Ghana | Africa | 20.4 | 8.5 | 14.4 | 13.5 | 19.1 | 4.9 | 50.0 | 22.9 |
| Kisumu | Kenya | Africa | 46.4 | 46.5 | 66.8 | 68.7 | 39.2 | 38.0 | 47.8 | 46.7 |
| Cape Town | South Africa | Africa | 26.4 | 25.9 | 5.0 | 6.3 | 24.3 | 24.5 | 55.9 | 56.1 |
| Delhi | India | Asia | 57.5 | 31.2 | 50.7 | 49.3 | 59.5 | 20.0 | 77.5 | 52.5 |
| Melbourne | Australia | Australia | 23.4 | 21.2 | 21.8 | 22.5 | 20.5 | 19.1 | 35.4 | 27.2 |
| London | England | Europe | 26.4 | 24.6 | 43.4 | 44.1 | 18.4 | 16.6 | 32.6 | 24.7 |
| Berlin | Germany | Europe | 19.4 | 16.7 | 12.4 | 13.4 | 18.4 | 15.1 | 25.2 | 23.5 |
| Cologne | Germany | Europe | 19.4 | 16.7 | 14.2 | 19.3 | 17.3 | 14.3 | 27.0 | 21.4 |
| Hamburg | Germany | Europe | 18.2 | 17.3 | 16.3 | 14.8 | 15.3 | 16.5 | 25.7 | 21.3 |
| Munich | Germany | Europe | 17.0 | 14.4 | 12.8 | 13.2 | 14.6 | 12.8 | 24.6 | 20.0 |
| Zurich | Switzerland | Europe | 11.0 | 8.4 | 10.0 | 7.3 | 7.6 | 7.5 | 20.0 | 11.6 |
| Buenos Aires | Argentina | Latin America | 37.9 | 33.5 | 39.4 | 40.8 | 31.1 | 25.9 | 58.1 | 48.7 |
| Sao Paulo | Brazil | Latin America | 32.0 | 23.1 | 21.0 | 22.9 | 28.6 | 18.3 | 62.1 | 48.6 |
| Santiago | Chile | Latin America | 23.2 | 19.5 | 27.2 | 27.9 | 18.9 | 15.2 | 33.3 | 24.3 |
| Bogota | Colombia | Latin America | 34.9 | 33.1 | 42.2 | 42.4 | 29.9 | 28.1 | 44.1 | 37.7 |
| Mexico City | Mexico | Latin America | 26.7 | 23.6 | 36.7 | 36.4 | 19.4 | 15.9 | 44.1 | 35.8 |
| Chicago | USA | North America | 12.7 | 11.1 | 12.7 | 12.4 | 9.5 | 8.3 | 22.4 | 19.1 |
| Los Angeles | USA | North America | 25.2 | 25.4 | 20.4 | 23.5 | 22.0 | 23.6 | 44.6 | 37.7 |
| New York City | USA | North America | 17.6 | 15.4 | 16.7 | 13.1 | 13.5 | 13.8 | 39.5 | 30.3 |

**Table A4: Sample size (number of person by gender and age group)**

| **City** | **Country** | **Region** | **All age groups combined** | | **Children** | | **Working age group** | | **Older adults** | |
| --- | --- | --- | --- | --- | --- | --- | --- | --- | --- | --- |
|  |  |  | **Female** | **Male** | **Female** | **Male** | **Female** | **Male** | **Female** | **Male** |
| Accra | Ghana | Africa | 485 | 425 | 90 | 104 | 361 | 286 | 34 | 35 |
| Kisumu | Kenya | Africa | 3155 | 2612 | 783 | 705 | 2255 | 1825 | 46 | 45 |
| Cape Town | South Africa | Africa | 3714 | 3430 | 517 | 521 | 2477 | 2274 | 556 | 451 |
| Delhi | India | Asia | 2962 | 3722 | 816 | 1086 | 2005 | 2452 | 130 | 168 |
| Melbourne | Australia | Australia | 25143 | 36178 | 5173 | 8114 | 14838 | 21057 | 5132 | 7007 |
| London | England | Europe | 30213 | 27464 | 7141 | 7167 | 17429 | 15582 | 5643 | 4715 |
| Berlin | Germany | Europe | 2081 | 2018 | 249 | 278 | 1130 | 1031 | 695 | 705 |
| Cologne | Germany | Europe | 3139 | 3085 | 404 | 433 | 1750 | 1613 | 975 | 1030 |
| Hamburg | Germany | Europe | 8697 | 8311 | 1047 | 1061 | 4552 | 4162 | 3083 | 3070 |
| Munich | Germany | Europe | 8741 | 8439 | 1069 | 1202 | 4819 | 4493 | 2841 | 2736 |
| Zurich | Switzerland | Europe | 2741 | 2712 | 366 | 400 | 1608 | 1619 | 767 | 693 |
| Buenos Aires | Argentina | Latin America | 35889 | 34432 | 9350 | 10157 | 20080 | 19135 | 6449 | 5121 |
| Sao Paulo | Brazil | Latin America | 12898 | 11636 | 2706 | 2916 | 8088 | 7251 | 2104 | 1469 |
| Santiago | Chile | Latin America | 31679 | 28375 | 6562 | 6921 | 19166 | 17086 | 5951 | 4368 |
| Bogota | Colombia | Latin America | 27633 | 24540 | 5385 | 5721 | 17115 | 14925 | 5133 | 3894 |
| Mexico City | Mexico | Latin America | 103296 | 96821 | 24208 | 25846 | 65505 | 59944 | 13583 | 11031 |
| Chicago | USA | North America | 17125 | 15218 | 3027 | 3292 | 9118 | 8150 | 4980 | 3776 |
| Los Angeles | USA | North America | 10790 | 10325 | 1714 | 1851 | 7163 | 6889 | 1440 | 1286 |
| New York City | USA | North America | 6410 | 5297 | 997 | 1071 | 4447 | 3521 | 853 | 636 |

**Table A5: Mode share of all age groups combined**

|  |  |  | **All age groups combined** | | | | | | | | | |
| --- | --- | --- | --- | --- | --- | --- | --- | --- | --- | --- | --- | --- |
| **City** | **Country** | **Region** | **Walking (%)** | | **Cycling (%)** | | **Public Transport (%)** | | **Car (%)** | | **Motorcycles (%)** | |
|  |  |  | **Female** | **Male** | **Female** | **Male** | **Female** | **Male** | **Female** | **Male** | **Female** | **Male** |
| Accra | Ghana | Africa | 60.6 | 56.4 | 0.1 | 0.8 | 28 | 23.6 | 6 | 10.5 | - | - |
| Kisumu | Kenya | Africa | 50.5 | 38.3 | 2.1 | 7 | 23.5 | 24.1 | 3.4 | 6.6 | 15 | 18.5 |
| Cape Town | South Africa | Africa | 30.2 | 29.2 | 0.1 | 0.4 | 32.8 | 27.3 | 33.3 | 39.3 | 0.1 | 0.6 |
| Delhi | India | Asia | 66.2 | 39.9 | 1.1 | 6.9 | 17.3 | 24 | 3.9 | 9.6 | 2.9 | 14.8 |
| Melbourne | Australia | Australia | 17 | 16.3 | 1.2 | 2.3 | 8.8 | 8.8 | 72.3 | 71.5 | 0 | 0.4 |
| London | England | Europe | 33.7 | 29.3 | 1.3 | 4 | 27.4 | 27.2 | 36.3 | 37.5 | 0.1 | 0.7 |
| Berlin | Germany | Europe | 26.8 | 24.1 | 15 | 13.2 | 23.7 | 19.7 | 28.4 | 33.6 | 0.4 | 0.5 |
| Cologne | Germany | Europe | 26 | 22.9 | 14.4 | 15 | 14.4 | 13.7 | 35.9 | 38.2 | 0.8 | 1.1 |
| Hamburg | Germany | Europe | 26.9 | 23.1 | 13.9 | 13.4 | 18.8 | 17.7 | 32.4 | 35.9 | 0.4 | 1.2 |
| Munich | Germany | Europe | 23.4 | 21.6 | 16.8 | 15.8 | 21.9 | 19.1 | 29.3 | 34 | 0.6 | 1.6 |
| Zurich | Switzerland | Europe | 37.1 | 32.1 | 5.7 | 6.9 | 17.7 | 14.5 | 37.6 | 42.5 | 0.4 | 1.4 |
| Buenos Aires | Argentina | Latin America | 32.1 | 22.3 | 2.2 | 4.3 | 47.7 | 41.7 | 14.1 | 27 | 0.3 | 2.3 |
| Sao Paulo | Brazil | Latin America | 34.8 | 28.1 | 0.2 | 1.1 | 38.2 | 32.5 | 25.8 | 33.6 | 0.6 | 4.2 |
| Santiago | Chile | Latin America | 33.9 | 24 | 2.5 | 5.1 | 34.1 | 31.1 | 23.9 | 33 | - | - |
| Bogota | Colombia | Latin America | 41 | 29.1 | 2.5 | 9 | 38.3 | 35 | 10.4 | 14.5 | 2.3 | 7.8 |
| Mexico City | Mexico | Latin America | 40.3 | 23.3 | 1 | 3.2 | 37.8 | 45.3 | 14.8 | 23.1 | 1.1 | 2.2 |
| Chicago | USA | North America | 11.1 | 10.3 | 0.5 | 1.3 | 6.3 | 6.9 | 80.9 | 80.3 | - | - |
| Los Angeles | USA | North America | 13.2 | 12 | 0.7 | 2 | 6 | 6 | 79.4 | 79 | 0.1 | 0.3 |
| New York City | USA | North America | 31.8 | 30.2 | 0.7 | 1.8 | 32 | 30.4 | 32.4 | 35.4 | - | - |

**Table A6: Number of trips of all age groups combined**

|  |  |  | **All age groups combined** | | | | | | | | | |
| --- | --- | --- | --- | --- | --- | --- | --- | --- | --- | --- | --- | --- |
|  |  |  | **Walking** | | **Cycling** | | **Public Transport** | | **Car** | | **Motorcycles** | |
| **City** | **Country** | **Region** | **Female** | **Male** | **Female** | **Male** | **Female** | **Male** | **Female** | **Male** | **Female** | **Male** |
| Accra | Ghana | Africa | 789 | 862 | 1 | 12 | 364 | 360 | 78 | 160 | 32 | 46 |
| Kisumu | Kenya | Africa | 2536 | 1625 | 105 | 295 | 1180 | 1021 | 172 | 282 | 24 | 114 |
| Cape Town | South Africa | Africa | 2025 | 1797 | 10 | 27 | 2199 | 1680 | 2237 | 2421 | 86 | 148 |
| Delhi | India | Asia | 1650 | 1869 | 23 | 267 | 489 | 1092 | 174 | 742 | 753 | 783 |
| Melbourne | Australia | Australia | 11052 | 14949 | 693 | 2049 | 4680 | 6866 | 51434 | 72141 | 208 | 476 |
| London | England | Europe | 23732 | 17802 | 974 | 2330 | 17971 | 15552 | 29584 | 26720 | 28 | 322 |
| Berlin | Germany | Europe | 1568 | 1359 | 814 | 755 | 1157 | 913 | 2113 | 2424 | 875 | 2693 |
| Cologne | Germany | Europe | 2329 | 2079 | 1253 | 1250 | 1255 | 996 | 3849 | 4272 | 122 | 321 |
| Hamburg | Germany | Europe | 6500 | 5479 | 3699 | 3516 | 4142 | 3576 | 9987 | 11056 | 58 | 444 |
| Munich | Germany | Europe | 6062 | 5646 | 4565 | 4364 | 5417 | 4543 | 8659 | 9744 | 40 | 138 |
| Zurich | Switzerland | Europe | 3341 | 3074 | 482 | 624 | 1461 | 1263 | 3655 | 4066 | - | - |
| Buenos Aires | Argentina | Latin America | 16549 | 11086 | 1468 | 2490 | 24920 | 21217 | 7038 | 13614 | 5 | 40 |
| Sao Paulo | Brazil | Latin America | 7942 | 6562 | 30 | 222 | 8558 | 7334 | 6622 | 8306 | - | - |
| Santiago | Chile | Latin America | 21116 | 12711 | 982 | 2888 | 22889 | 19737 | 11473 | 14964 | - | - |
| Bogota | Colombia | Latin America | 17533 | 11485 | 984 | 3040 | 16495 | 13276 | 5491 | 6635 | 239 | 1290 |
| Mexico City | Mexico | Latin America | 71203 | 36779 | 1891 | 5068 | 66088 | 70714 | 24512 | 34941 | 128 | 870 |
| Chicago | USA | North America | 3788 | 3174 | 203 | 430 | 2042 | 1999 | 37946 | 31207 | 132 | 1411 |
| Los Angeles | USA | North America | 3599 | 3132 | 245 | 590 | 1405 | 1313 | 27059 | 24548 | 3248 | 5523 |
| New York City | USA | North America | 6334 | 4840 | 169 | 327 | 6019 | 4847 | 6170 | 5554 | - | - |

**Table A7: Mode share of children**

|  |  |  | **Children** | | | | | | | | | |
| --- | --- | --- | --- | --- | --- | --- | --- | --- | --- | --- | --- | --- |
| **City** | **Country** | **Region** | **Walking (%)** | | **Cycling (%)** | | **Public Transport (%)** | | **Car (%)** | | **Motorcycles (%)** | |
|  |  |  | **Female** | **Male** | **Female** | **Male** | **Female** | **Male** | **Female** | **Male** | **Female** | **Male** |
| Accra | Ghana | Africa | 86.1 | 84.4 | - | 0.7 | 10.8 | 11.3 | 2.4 | 2.7 | - | - |
| Kisumu | Kenya | Africa | 72.9 | 74.5 | 1.6 | 4.3 | 10 | 10.3 | 2.1 | 1 | 10.3 | 6.5 |
| Cape Town | South Africa | Africa | 53.6 | 53.3 | 0 | 0 | 20.9 | 21.9 | - | 0.3 | 24.1 | 23.2 |
| Delhi | India | Asia | 72.4 | 71.6 | 1.9 | 2.3 | 12.1 | 14.1 | 1.5 | 2.1 | 1 | 3 |
| Melbourne | Australia | Australia | 17.6 | 17.4 | 1.5 | 2.2 | 8.1 | 8.4 | 72.5 | 71.6 | - | 0 |
| London | England | Europe | 36.9 | 36.5 | 1.1 | 3.3 | 27.2 | 24.7 | 34.2 | 34.6 | - | 0.1 |
| Berlin | Germany | Europe | 28.3 | 28.3 | 22.6 | 22.5 | 16.8 | 14.2 | 24.3 | 26.1 | 0.5 | 0.5 |
| Cologne | Germany | Europe | 29.7 | 25.6 | 18 | 15.7 | 12.1 | 12 | 32 | 35 | 0.2 | 0.5 |
| Hamburg | Germany | Europe | 33.6 | 28.6 | 19.4 | 23 | 12.2 | 11.4 | 28.7 | 27 | 0.6 | 0.6 |
| Munich | Germany | Europe | 29.1 | 30.7 | 21.1 | 20.9 | 15.4 | 13 | 26.4 | 28.5 | 0.2 | 0.1 |
| Zurich | Switzerland | Europe | 51.8 | 51.4 | 7 | 10.8 | 15.9 | 13.3 | 21.9 | 15.7 | 0.8 | 1.1 |
| Buenos Aires | Argentina | Latin America | 48.3 | 49.2 | 1.8 | 2.4 | 32.6 | 32.5 | 14.5 | 13.1 | 0.3 | 0.3 |
| Sao Paulo | Brazil | Latin America | 52.6 | 54.9 | 0.2 | 0.5 | 31.4 | 30.4 | 15.4 | 13.9 | 0.2 | 0.2 |
| Santiago | Chile | Latin America | 40 | 42.9 | 3.6 | 2.4 | 20.5 | 19.7 | 20.7 | 20.2 | - | - |
| Bogota | Colombia | Latin America | 57.7 | 54.8 | 3 | 7 | 29.4 | 28.3 | 5.1 | 5.6 | 1.5 | 1.6 |
| Mexico City | Mexico | Latin America | 53.5 | 54.5 | 1.1 | 1.8 | 28.5 | 28.2 | 11.6 | 10.9 | 1.5 | 1.5 |
| Chicago | USA | North America | 14.7 | 13.8 | 0.8 | 1.8 | 10.1 | 11 | 74 | 72.8 | - | - |
| Los Angeles | USA | North America | 17.7 | 20.6 | 1.1 | 1.5 | 6.8 | 6.7 | 73.7 | 70.5 | - | 0 |
| New York City | USA | North America | 35 | 38.9 | 0.7 | 1.5 | 33.6 | 29.1 | 30 | 29 | - | - |

**Table A8: Number of trips of children**

|  |  |  | **Children** | | | | | | | | | |
| --- | --- | --- | --- | --- | --- | --- | --- | --- | --- | --- | --- | --- |
|  |  |  | **Walking** | | **Cycling** | | **Public Transport** | | **Car** | | **Motorcycles** | |
| **City** | **Country** | **Region** | **Female** | **Male** | **Female** | **Male** | **Female** | **Male** | **Female** | **Male** | **Female** | **Male** |
| Accra | Ghana | Africa | 34 | 0 | 0 | 2 | 3 | - | 2 | 179 | 219 | 524 |
| Kisumu | Kenya | Africa | 433 | 11 | 25 | 68 | 60 | 14 | 6 | - | 4 | 1951 |
| Cape Town | South Africa | Africa | 556 | 5664 | 6187 | 221 | 228 | - | 3 | 4 | 4 | 1335 |
| Delhi | India | Asia | 732 | 13 | 25 | 124 | 198 | 24 | 40 | 70 | 38 | 1066 |
| Melbourne | Australia | Australia | 3059 | 176 | 474 | 803 | 1295 | 8967 | 13774 | 8 | 4 | 7153 |
| London | England | Europe | 4054 | 154 | 360 | 2679 | 2459 | 4258 | 4267 | - | 2 | 15838 |
| Berlin | Germany | Europe | 111 | 247 | 254 | 46 | 52 | 148 | 159 | 405 | 463 | 771 |
| Cologne | Germany | Europe | 290 | 198 | 209 | 164 | 147 | 332 | 364 | 10 | 21 | 1314 |
| Hamburg | Germany | Europe | 741 | 620 | 697 | 334 | 318 | 873 | 816 | - | 14 | 3486 |
| Munich | Germany | Europe | 930 | 642 | 733 | 492 | 455 | 834 | 983 | 15 | 21 | 3307 |
| Zurich | Switzerland | Europe | 741 | 99 | 171 | 214 | 191 | 299 | 257 | - | - | 1828 |
| Buenos Aires | Argentina | Latin America | 4232 | 3670 | 3810 | 269 | 293 | 285 | 405 | 254 | 242 | 9099 |
| Sao Paulo | Brazil | Latin America | 2740 | 7 | 28 | 1523 | 1528 | 855 | 833 | - | - | 4879 |
| Santiago | Chile | Latin America | 4799 | 152 | 276 | 2269 | 2478 | 1762 | 1753 | - | - | 12205 |
| Bogota | Colombia | Latin America | 2078 | 182 | 182 | 242 | 210 | 157 | 407 | 1622 | 1571 | 10572 |
| Mexico City | Mexico | Latin America | 17362 | 18745 | 390 | 664 | 9092 | 9655 | 3475 | 3530 | 10 | 11 |
| Chicago | USA | North America | 686 | 37 | 107 | 630 | 710 | 4731 | 4779 | 17 | 54 | 2299 |
| Los Angeles | USA | North America | 820 | 60 | 70 | 234 | 252 | 3628 | 3667 | 631 | 714 | 2411 |
| New York City | USA | North America | 967 | 15 | 46 | 785 | 770 | 666 | 841 | - | - | 4685 |

**Table A9: Mode share of working age group**

|  |  |  | **Working age** | | | | | | | | | |
| --- | --- | --- | --- | --- | --- | --- | --- | --- | --- | --- | --- | --- |
| **City** | **Country** | **Region** | **Walking (%)** | | **Cycling (%)** | | **Public Transport (%)** | | **Car (%)** | | **Motorcycles (%)** | |
|  |  |  | **Female** | **Male** | **Female** | **Male** | **Female** | **Male** | **Female** | **Male** | **Female** | **Male** |
| Accra | Ghana | Africa | 54 | 49.5 | 0.1 | 0.9 | 32.2 | 26 | 6.9 | 12.3 | - | - |
| Kisumu | Kenya | Africa | 46.7 | 32.3 | 2.2 | 7.2 | 25.5 | 26.4 | 3.7 | 7.3 | 16 | 20.8 |
| Cape Town | South Africa | Africa | 27 | 25.2 | 0.2 | 0.4 | 36.5 | 29.9 | 32.9 | 40.7 | 0.1 | 0.8 |
| Delhi | India | Asia | 63.2 | 29.1 | 0.5 | 8.6 | 20 | 27.5 | 5.1 | 12 | 3.8 | 19 |
| Melbourne | Australia | Australia | 16.6 | 15.6 | 1.2 | 2.6 | 9.6 | 10 | 71.9 | 70.5 | 0 | 0.5 |
| London | England | Europe | 33.4 | 27.4 | 1.4 | 4.6 | 27.6 | 28.5 | 36 | 37 | 0.1 | 0.9 |
| Berlin | Germany | Europe | 21.1 | 21.2 | 15.9 | 11.7 | 26.7 | 21.4 | 29.5 | 35.1 | 0.5 | 0.5 |
| Cologne | Germany | Europe | 23.3 | 20.7 | 15.8 | 16.8 | 15.2 | 15.4 | 36.1 | 37.4 | 1.1 | 1.1 |
| Hamburg | Germany | Europe | 23.8 | 21.4 | 13.9 | 12.4 | 21 | 20.7 | 32.6 | 35.7 | 0.3 | 1 |
| Munich | Germany | Europe | 20.9 | 19 | 16.9 | 15.5 | 23 | 20.9 | 29.5 | 34 | 0.6 | 1.8 |
| Zurich | Switzerland | Europe | 32.4 | 26.9 | 6.3 | 7.1 | 19.6 | 16.3 | 39.9 | 46.1 | 0.4 | 1.7 |
| Buenos Aires | Argentina | Latin America | 26.7 | 12.3 | 2.4 | 4.8 | 52.9 | 46 | 14.4 | 31.6 | 0.4 | 3.4 |
| Sao Paulo | Brazil | Latin America | 30.1 | 19.6 | 0.2 | 1.3 | 40.5 | 34 | 28.2 | 38.6 | 0.8 | 5.9 |
| Santiago | Chile | Latin America | 30 | 17.8 | 2.6 | 6 | 38.5 | 34.8 | 25.7 | 36.1 | - | - |
| Bogota | Colombia | Latin America | 35.2 | 20.3 | 2.9 | 10.7 | 42.5 | 37.2 | 11 | 16 | 2.9 | 10.9 |
| Mexico City | Mexico | Latin America | 36.5 | 13 | 1.1 | 3.5 | 41.3 | 52 | 15.5 | 26.2 | 1.1 | 2.6 |
| Chicago | USA | North America | 10.7 | 9.5 | 0.4 | 1.2 | 5.7 | 6.1 | 81.9 | 81.7 | - | - |
| Los Angeles | USA | North America | 12.5 | 9.7 | 0.7 | 2.2 | 6 | 6 | 80.4 | 81 | 0.1 | 0.4 |
| New York City | USA | North America | 31.1 | 27.6 | 0.8 | 1.9 | 32.3 | 31.7 | 32.5 | 36.2 | - | - |

**Table A10: Number of trips of working age group**

|  |  |  | **Working age** | | | | | | | | | |
| --- | --- | --- | --- | --- | --- | --- | --- | --- | --- | --- | --- | --- |
|  |  |  | **Walking** | | **Cycling** | | **Public Transport** | | **Car** | | **Motorcycles** | |
| **City** | **Country** | **Region** | **Female** | **Male** | **Female** | **Male** | **Female** | **Male** | **Female** | **Male** | **Female** | **Male** |
| Accra | Ghana | Africa | 561 | 1 | 10 | 313 | 295 | 67 | 140 | 21 | 25 | 20 |
| Kisumu | Kenya | Africa | 1151 | 91 | 255 | 1065 | 940 | 156 | 259 | 21 | 94 | 35 |
| Cape Town | South Africa | Africa | 1123 | 10 | 18 | 1804 | 1334 | 1624 | 1812 | 71 | 86 | 119 |
| Delhi | India | Asia | 1033 | 8 | 238 | 346 | 863 | 140 | 674 | 667 | 740 | 17 |
| Melbourne | Australia | Australia | 8843 | 447 | 1364 | 3308 | 4842 | 33627 | 44833 | 135 | 321 | 2030 |
| London | England | Europe | 10408 | 747 | 1794 | 12383 | 10666 | 20178 | 17134 | 28 | 270 | 3695 |
| Berlin | Germany | Europe | 668 | 519 | 404 | 749 | 567 | 1292 | 1413 | 769 | 2523 | 301 |
| Cologne | Germany | Europe | 992 | 849 | 772 | 835 | 647 | 2411 | 2411 | 54 | 166 | 252 |
| Hamburg | Germany | Europe | 2746 | 2257 | 1959 | 2766 | 2417 | 5940 | 6015 | 58 | 380 | 1028 |
| Munich | Germany | Europe | 2880 | 2887 | 2519 | 3450 | 2883 | 5311 | 5587 | 23 | 101 | 1944 |
| Zurich | Switzerland | Europe | 1555 | 324 | 379 | 1001 | 903 | 2496 | 2824 | - | - | 878 |
| Buenos Aires | Argentina | Latin America | 3721 | 1112 | 1710 | 18084 | 14869 | 4747 | 10261 | 5 | 37 | 2780 |
| Sao Paulo | Brazil | Latin America | 3359 | 23 | 186 | 6315 | 5320 | 5008 | 6382 | - | - | 509 |
| Santiago | Chile | Latin America | 5581 | 791 | 2200 | 17238 | 14471 | 8159 | 10866 | - | - | 4252 |
| Bogota | Colombia | Latin America | 5561 | 810 | 2387 | 12461 | 9359 | 4071 | 4819 | 182 | 1206 | 1925 |
| Mexico City | Mexico | Latin America | 46417 | 14057 | 1458 | 3767 | 51955 | 55370 | 18478 | 27165 | 118 | 843 |
| Chicago | USA | North America | 1887 | 131 | 264 | 1085 | 1066 | 22438 | 17413 | 107 | 1323 | 327 |
| Los Angeles | USA | North America | 1916 | 162 | 451 | 995 | 935 | 19598 | 17500 | 2352 | 4603 | 299 |
| New York City | USA | North America | 3318 | 146 | 256 | 4632 | 3659 | 4726 | 3818 | - | - | 621 |

**Table A11: Mode share of older adults**

|  |  |  | **Older adults** | | | | | | | | | |
| --- | --- | --- | --- | --- | --- | --- | --- | --- | --- | --- | --- | --- |
| **City** | **Country** | **Region** | **Walking (%)** | | **Cycling (%)** | | **Public Transport (%)** | | **Car (%)** | | **Motorcycles (%)** | |
|  |  |  | **Female** | **Male** | **Female** | **Male** | **Female** | **Male** | **Female** | **Male** | **Female** | **Male** |
| Accra | Ghana | Africa | 40.9 | 50.5 | 0 | 0 | 45.5 | 33.3 | 9.1 | 12.9 | - | - |
| Kisumu | Kenya | Africa | 41.7 | 38.9 | 0 | 0 | 38.1 | 23.6 | 1.2 | 12.5 | 2.4 | 20.8 |
| Cape Town | South Africa | Africa | 17.8 | 13.1 | - | 1 | 24.9 | 15.8 | 46.2 | 59.9 | - | 0.7 |
| Delhi | India | Asia | 53.7 | 56.2 | - | 0.6 | 25 | 16.1 | 8.1 | 9.3 | 8.2 | 8.6 |
| Melbourne | Australia | Australia | 18.2 | 17.7 | 0.8 | 1.3 | 6 | 4.6 | 74 | 75.4 | - | 0.3 |
| London | England | Europe | 31.5 | 30.1 | 0.6 | 1.8 | 26.6 | 23.2 | 39.6 | 43.4 | - | 0.5 |
| Berlin | Germany | Europe | 40.1 | 31.1 | 8.1 | 11.2 | 20.2 | 18.2 | 28.3 | 34.2 | 0.1 | 0.5 |
| Cologne | Germany | Europe | 32.5 | 28.7 | 7.1 | 8.8 | 13.4 | 8.9 | 38.1 | 43 | 0.5 | 1.5 |
| Hamburg | Germany | Europe | 31.9 | 25.1 | 10.7 | 9.6 | 16.3 | 12.1 | 34.1 | 43.4 | 0.6 | 2.3 |
| Munich | Germany | Europe | 27.6 | 23.3 | 14.1 | 13.1 | 22.2 | 17.3 | 30.6 | 38.3 | 0.7 | 1.9 |
| Zurich | Switzerland | Europe | 44.3 | 38.2 | 3 | 3.6 | 12.4 | 9 | 39.2 | 47.3 | 0.1 | 0.6 |
| Buenos Aires | Argentina | Latin America | 31.3 | 20.8 | 1.1 | 5.9 | 47.1 | 37 | 11.3 | 31.6 | 0 | 0.3 |
| Sao Paulo | Brazil | Latin America | 25.5 | 24.2 | - | 0.4 | 38.2 | 25.8 | 34.2 | 48.1 | - | 0.9 |
| Santiago | Chile | Latin America | 43.8 | 27.4 | 0.3 | 4.1 | 32.6 | 29.2 | 20.2 | 36.8 | - | - |
| Bogota | Colombia | Latin America | 49.9 | 35.1 | 0.2 | 4.2 | 28.3 | 33.9 | 13 | 19.7 | 0.2 | 1.4 |
| Mexico City | Mexico | Latin America | 43.9 | 25.5 | 0.2 | 4 | 29.7 | 36.5 | 16 | 28.2 | 0.9 | 0.8 |
| Chicago | USA | North America | 8.8 | 7.8 | 0.3 | 0.7 | 4.1 | 3.6 | 85.5 | 86.7 | - | - |
| Los Angeles | USA | North America | 11.1 | 11.1 | 0.2 | 1.5 | 4.8 | 5.3 | 82 | 80.9 | 0.1 | 0.5 |
| New York City | USA | North America | 30 | 28.3 | 0.3 | 1.4 | 26.7 | 23.5 | 36.3 | 44.1 | - | - |

**Table A12: Number of trips of older adults**

|  |  |  | **Older adults** | | | | | | | | | |
| --- | --- | --- | --- | --- | --- | --- | --- | --- | --- | --- | --- | --- |
|  |  |  | **Walking** | | **Cycling** | | **Public Transport** | | **Car** | | **Motorcycles** | |
| **City** | **Country** | **Region** | **Female** | **Male** | **Female** | **Male** | **Female** | **Male** | **Female** | **Male** | **Female** | **Male** |
| Accra | Ghana | Africa | 31 | 0 | 0 | 2 | 3 | 0 | 0 | 638 | 791 |  |
| Kisumu | Kenya | Africa | 28 | 2173 | 1978 | 32 | 17 | 1 | 9 | 3 | 14 | 20.8 |
| Cape Town | South Africa | Africa | 65 | 1784 | 1167 | 53 | 39 | - | 4 | 1100 | 1479 | 0.7 |
| Delhi | India | Asia | 27 | 676 | 796 | 3 | 11 | - | 2 | 2 | 15 | 8.6 |
| Melbourne | Australia | Australia | 3047 | 70 | 211 | 569 | 729 | 8840 | 13534 | 65 | 151 | 0.3 |
| London | England | Europe | 3340 | 73 | 176 | 2909 | 2427 | 5148 | 5319 | - | 50 | 0.5 |
| Berlin | Germany | Europe | 235 | 18 | 47 | 83 | 116 | 143 | 192 | 1015 | 1353 | 0.5 |
| Cologne | Germany | Europe | 200 | 808 | 601 | 184 | 225 | 204 | 267 | 3160 | 4210 | 1.5 |
| Hamburg | Germany | Europe | 832 | 42 | 102 | 536 | 616 | 822 | 855 | - | 50 | 2.3 |
| Munich | Germany | Europe | 1832 | 1035 | 1110 | 1464 | 1203 | 2504 | 3168 | 2 | 16 | 1.9 |
| Zurich | Switzerland | Europe | 778 | 59 | 74 | 246 | 169 | 860 | 985 | - | - | 0.6 |
| Buenos Aires | Argentina | Latin America | 2107 | 3291 | 2114 | 500 | 241 | 71 | 375 | 221 | 246 | 0.3 |
| Sao Paulo | Brazil | Latin America | 463 | - | 8 | 720 | 486 | 759 | 1091 | - | - | 0.9 |
| Santiago | Chile | Latin America | 2331 | 39 | 412 | 3382 | 2788 | 1552 | 2345 | - | - |  |
| Bogota | Colombia | Latin America | 1839 | 608 | 509 | 658 | 356 | 17 | 246 | 666 | 1770 | 1.4 |
| Mexico City | Mexico | Latin America | 7424 | 3977 | 43 | 637 | 5041 | 5689 | 2559 | 4246 | - | 16 |
| Chicago | USA | North America | 223 | 85 | 54 | 95 | 62 | 35 | 59 | 10 | 28 |  |
| Los Angeles | USA | North America | 317 | 8 | 49 | 110 | 104 | 2687 | 2707 | 265 | 206 | 0.5 |
| New York City | USA | North America | 487 | 8 | 24 | 486 | 352 | 681 | 818 | - | - |  |

**Table A13: Mode share of work trips of all age groups**

| **City** | **Country** | **Region** | **Walking (%)** | | **Cycling (%)** | | **Public Transport (%)** | | **Car (%)** | | **Motorcycles (%)** | |
| --- | --- | --- | --- | --- | --- | --- | --- | --- | --- | --- | --- | --- |
|  |  |  | **Female** | **Male** | **Female** | **Male** | **Female** | **Male** | **Female** | **Male** | **Female** | **Male** |
| Accra | Ghana | Africa | 47.3 | 30.5 | 0.3 | 1.1 | 36.3 | 39.2 | 5.1 | 13.0 | 0.0 | 0.0 |
| Kisumu | Kenya | Africa | 37.1 | 27.6 | 1.9 | 7.0 | 27.0 | 23.6 | 8.8 | 9.4 | 20.4 | 25.0 |
| Cape Town | South Africa | Africa | 15.3 | 15.6 | 0.4 | 0.8 | 41.7 | 31.5 | 41.1 | 49.9 | 0.1 | 1.2 |
| Delhi | India | Asia | 37.6 | 25.7 | 2.2 | 10.0 | 34.4 | 28.8 | 10.4 | 12.6 | 8.9 | 19.7 |
| Melbourne | Australia | Australia | 8.5 | 7.0 | 1.6 | 2.6 | 19.1 | 15.0 | 70.2 | 74.0 | 0.1 | 0.8 |
| London | England | Europe | 16.6 | 11.8 | 2.6 | 6.2 | 49.1 | 42.9 | 30.3 | 36.5 | 0.4 | 1.7 |
| Berlin | Germany | Europe | 6.5 | 7.5 | 19.0 | 16.3 | 40.5 | 33.7 | 26.1 | 30.2 | 0.3 | 1.1 |
| Cologne | Germany | Europe | 10.0 | 10.0 | 17.5 | 18.3 | 25.3 | 26.2 | 36.2 | 35.1 | 1.4 | 1.5 |
| Hamburg | Germany | Europe | 7.1 | 6.2 | 16.9 | 14.6 | 35.6 | 32.3 | 29.7 | 33.4 | 0.4 | 1.3 |
| Munich | Germany | Europe | 6.0 | 6.7 | 18.0 | 19.4 | 40.7 | 32.0 | 25.7 | 30.6 | 0.6 | 2.0 |
| Zurich | Switzerland | Europe | 23.1 | 19.3 | 8.1 | 8.9 | 33.5 | 24.3 | 34.2 | 44.7 | 0.6 | 2.2 |
| Buenos Aires | Argentina | Latin America | 13.6 | 9.3 | 2.2 | 5.6 | 67.9 | 49.5 | 13.5 | 30.4 | 0.3 | 3.5 |
| Sao Paulo | Brazil | Latin America | 23.7 | 18.2 | 0.2 | 1.6 | 50.9 | 36.7 | 23.9 | 36.6 | 1.0 | 6.2 |
| Santiago | Chile | Latin America | 14.3 | 10.2 | 2.4 | 6.1 | 56.8 | 42.6 | 22.1 | 35.3 | 0.0 | 0.0 |
| Bogota | Colombia | Latin America | 15.0 | 10.3 | 3.0 | 11.7 | 61.3 | 42.8 | 11.1 | 15.7 | 4.0 | 13.9 |
| Mexico City | Mexico | Latin America | 15.4 | 11.3 | 1.0 | 3.7 | 59.0 | 53.5 | 19.6 | 26.6 | 0.8 | 2.4 |
| Chicago | USA | North America | 8.5 | 6.4 | 0.6 | 1.2 | 11.6 | 8.9 | 76.7 | 81.4 | 0.0 | 0.0 |
| Los Angeles | USA | North America | 6.8 | 5.2 | 0.5 | 2.5 | 8.1 | 7.2 | 84.1 | 84.2 | 0.1 | 0.6 |
| New York City | USA | North America | 10.9 | 9.5 | 0.7 | 2.0 | 57.0 | 54.2 | 28.5 | 32.1 | 0.0 | 0.0 |

**Table A14: Number of work trips of all age groups**

|  |  |  | **Walk** | | **Bike** | | **Public transport** | | **Car** | | **Motorcycles** | |
| --- | --- | --- | --- | --- | --- | --- | --- | --- | --- | --- | --- | --- |
| **City** | **Country** | **Region** | **Female** | **Male** | **Female** | **Male** | **Female** | **Male** | **Female** | **Male** | **Female** | **Male** |
| Accra | Ghana | Africa | 167 | 134 | 1 | 5 | 128 | 172 | 18 | 57 | 0 | 0 |
| Kisumu | Kenya | Africa | 249 | 289 | 13 | 73 | 181 | 248 | 59 | 99 | 68 | 1169 |
| Cape Town | South Africa | Africa | 310 | 325 | 9 | 16 | 843 | 656 | 831 | 1040 | 2 | 15 |
| Delhi | India | Asia | 170 | 758 | 7 | 233 | 170 | 755 | 68 | 602 | 3 | 24 |
| Melbourne | Australia | Australia | 880 | 1407 | 140 | 556 | 1964 | 3261 | 8596 | 17626 | 44 | 262 |
| London | England | Europe | 1927 | 1708 | 328 | 908 | 5559 | 6219 | 4184 | 6626 | 17 | 53 |
| Berlin | Germany | Europe | 53 | 57 | 146 | 127 | 322 | 223 | 283 | 270 | 0 | 0 |
| Cologne | Germany | Europe | 110 | 97 | 200 | 214 | 280 | 273 | 536 | 702 | 50 | 846 |
| Hamburg | Germany | Europe | 245 | 214 | 573 | 592 | 1115 | 1024 | 1222 | 1462 | 25 | 27 |
| Munich | Germany | Europe | 243 | 262 | 697 | 786 | 1364 | 1202 | 1028 | 1367 | 7 | 167 |
| Zurich | Switzerland | Europe | 356 | 403 | 124 | 159 | 503 | 501 | 590 | 1058 | 12 | 52 |
| Buenos Aires | Argentina | Latin America | 1767 | 1908 | 380 | 1350 | 8876 | 10806 | 1778 | 6694 | 0 | 0 |
| Sao Paulo | Brazil | Latin America | 2237 | 2342 | 14 | 164 | 4478 | 4222 | 2503 | 4637 | 83 | 671 |
| Santiago | Chile | Latin America | 1696 | 1954 | 316 | 1591 | 8136 | 10438 | 2241 | 6089 | 39 | 113 |
| Bogota | Colombia | Latin America | 1617 | 1526 | 292 | 1295 | 5904 | 5310 | 1360 | 2340 | 0 | 0 |
| Mexico City | Mexico | Latin America | 8222 | 9927 | 515 | 3281 | 30755 | 45873 | 9806 | 22151 | 2 | 43 |
| Chicago | USA | North America | 518 | 577 | 43 | 95 | 597 | 654 | 6016 | 7581 | 367 | 1594 |
| Los Angeles | USA | North America | 267 | 283 | 32 | 121 | 308 | 337 | 4020 | 5142 | 137 | 262 |
| New York City | USA | North America | 344 | 298 | 31 | 87 | 1940 | 1789 | 968 | 1056 | 442 | 2066 |

**Table A15: Percentage contribution to total active travel time by different main modes for all age groups**

| **City** | **Country** | **Region** | **Active travel for public transport** | **Females** | | | **Males** | | |
| --- | --- | --- | --- | --- | --- | --- | --- | --- | --- |
|  |  |  |  | **Walk** | **Cycle** | **Public transport** | **Walk** | **Cycle** | **Public transport** |
| Accra | Ghana | Africa | harmonised | 81.1 | 0 | 17.8 | 79.5 | 1.2 | 19.3 |
| Kisumu | Kenya | Africa | harmonised | 76.2 | 3.7 | 18.4 | 69.6 | 10.9 | 19.5 |
| Cape Town | South Africa | Africa | harmonised | 65.6 | 0.4 | 31.8 | 69.1 | 1 | 29.9 |
| Delhi | India | Asia | reported | 90.5 | 1.5 | 6.6 | 61.2 | 21.3 | 16.4 |
| Melbourne | Australia | Australia | reported | 60.4 | 6.9 | 31.2 | 53.4 | 14.3 | 30.4 |
| London | England | Europe | reported | 62.2 | 3 | 30.7 | 51.4 | 10 | 34.5 |
| Berlin | Germany | Europe | harmonised | 47.5 | 23.1 | 27.4 | 46.3 | 27.7 | 26 |
| Cologne | Germany | Europe | harmonised | 57.9 | 22.9 | 16.6 | 50.3 | 30.3 | 19.3 |
| Hamburg | Germany | Europe | harmonised | 52.8 | 22.3 | 22.6 | 47.6 | 27.8 | 24.6 |
| Munich | Germany | Europe | harmonised | 46.3 | 25.9 | 25.7 | 43.5 | 30.5 | 26 |
| Zurich | Switzerland | Europe | reported | 75.1 | 8.1 | 14.5 | 71.3 | 12.5 | 14.2 |
| Buenos Aires | Argentina | Latin America | harmonised | 44 | 2.7 | 51.3 | 36.9 | 8.3 | 54.7 |
| Sao Paulo | Brazil | Latin America | reported | 48.6 | 0.3 | 42.2 | 44.2 | 3.4 | 39.8 |
| Santiago | Chile | Latin America | harmonised | 47.4 | 3.9 | 46.1 | 39.8 | 13.9 | 46.3 |
| Bogota | Colombia | Latin America | reported | 67.2 | 6.5 | 23.4 | 49.1 | 28 | 19.2 |
| Mexico City | Mexico | Latin America | reported | 63.9 | 1.8 | 33.2 | 43.5 | 8.2 | 47.2 |
| Chicago | USA | North America | reported | 72.6 | 4.1 | 20.5 | 66.7 | 10.5 | 20.2 |
| Los Angeles | USA | North America | reported | 70.8 | 5.6 | 22.3 | 59.5 | 18.7 | 20.6 |
| New York City | USA | North America | reported | 51 | 1.7 | 42.9 | 48.9 | 3.6 | 41.9 |

**Table A16: Percentage contribution to total active travel time by different main modes for children**

| **City** | **Country** | **Region** | **Active travel for public transport** | **Children (female)** | | | **Children (male)** | | |
| --- | --- | --- | --- | --- | --- | --- | --- | --- | --- |
|  |  |  |  | **Walk** | **Cycle** | **Public transport** | **Walk** | **Cycle** | **Public transport** |
| Accra | Ghana | Africa | harmonised | 93.7 | NA | 6.1 | 92.2 | 1.1 | 6.7 |
| Kisumu | Kenya | Africa | harmonised | 89.4 | 2.1 | 7.4 | 87.4 | 5.7 | 7 |
| Cape Town | South Africa | Africa | harmonised | 81.5 | NA | 17.2 | 84.1 | 0.2 | 15.7 |
| Delhi | India | Asia | reported | 93.5 | 1.3 | 4.2 | 89.2 | 2.6 | 6.1 |
| Melbourne | Australia | Australia | reported | 66.2 | 7.3 | 26.1 | 62.8 | 8.8 | 27.9 |
| London | England | Europe | reported | 66.5 | 1.8 | 27.7 | 62.4 | 6.3 | 27.3 |
| Berlin | Germany | Europe | harmonised | 47.2 | 31.9 | 19.1 | 44.6 | 36.5 | 18.9 |
| Cologne | Germany | Europe | harmonised | 61.1 | 23.5 | 13.4 | 54.4 | 30.6 | 15 |
| Hamburg | Germany | Europe | harmonised | 55.4 | 28.9 | 13.6 | 51.4 | 35.4 | 13.3 |
| Munich | Germany | Europe | harmonised | 57.2 | 24.9 | 16.3 | 53.5 | 30.5 | 16.1 |
| Zurich | Switzerland | Europe | reported | 74.5 | 11.4 | 12.6 | 73.7 | 14.6 | 10.8 |
| Buenos Aires | Argentina | Latin America | harmonised | 62.2 | 2.1 | 33.9 | 63.8 | 3 | 33.2 |
| Sao Paulo | Brazil | Latin America | reported | 75.8 | 0.3 | 19.3 | 78.1 | 0.7 | 17.2 |
| Santiago | Chile | Latin America | harmonised | 66.3 | 3.7 | 27.8 | 70.2 | 5.2 | 24.6 |
| Bogota | Colombia | Latin America | reported | 82.8 | 5.3 | 10.6 | 73.1 | 16.2 | 9.6 |
| Mexico City | Mexico | Latin America | reported | 79.4 | 1.7 | 18.1 | 78.6 | 2.8 | 17.8 |
| Chicago | USA | North America | reported | 84.5 | 3.2 | 11.6 | 83.8 | 7.7 | 7.6 |
| Los Angeles | USA | North America | reported | 80.9 | 4.4 | 13.8 | 76 | 10.2 | 13 |
| New York City | USA | North America | reported | 60.1 | 1.4 | 36.4 | 61.1 | 2.7 | 31.7 |

**Table A17: Percentage contribution to total active travel time by different main modes for working age group**

| **City** | **Country** | **Region** | **Active travel for public transport** | **Working age group (female)** | | | **Working age group (male)** | | |
| --- | --- | --- | --- | --- | --- | --- | --- | --- | --- |
|  |  |  |  | **Walk** | **Cycle** | **Public transport** | **Walk** | **Cycle** | **Public transport** |
| Accra | Ghana | Africa | harmonised | 77.8 | 0 | 20.8 | 75.1 | 1.3 | 23.6 |
| Kisumu | Kenya | Africa | harmonised | 73.4 | 4 | 20.7 | 64.8 | 12.1 | 23.1 |
| Cape Town | South Africa | Africa | harmonised | 63.1 | 0.5 | 34.3 | 64.9 | 0.8 | 34.3 |
| Delhi | India | Asia | reported | 88.7 | 1.3 | 8.4 | 49 | 29.7 | 21.3 |
| Melbourne | Australia | Australia | reported | 56.8 | 7.3 | 34.2 | 47.2 | 16.6 | 34 |
| London | England | Europe | reported | 60.6 | 3.5 | 31.9 | 47.2 | 11.9 | 37.4 |
| Berlin | Germany | Europe | harmonised | 37.6 | 26.6 | 33.7 | 40 | 29.2 | 30.9 |
| Cologne | Germany | Europe | harmonised | 51.4 | 27.4 | 18.6 | 42.7 | 33.8 | 23.5 |
| Hamburg | Germany | Europe | harmonised | 48.3 | 22.9 | 26.4 | 41.7 | 27.7 | 30.6 |
| Munich | Germany | Europe | harmonised | 40.4 | 27.9 | 29.4 | 37 | 32.4 | 30.7 |
| Zurich | Switzerland | Europe | reported | 71.2 | 9 | 17.3 | 64.7 | 14.3 | 18.4 |
| Buenos Aires | Argentina | Latin America | harmonised | 37.4 | 3.2 | 57.6 | 22.9 | 10.6 | 66.5 |
| Sao Paulo | Brazil | Latin America | reported | 41 | 0.3 | 48.9 | 30.7 | 4.8 | 49.1 |
| Santiago | Chile | Latin America | harmonised | 40.1 | 4.8 | 52.4 | 28.4 | 17.1 | 54.5 |
| Bogota | Colombia | Latin America | reported | 61 | 8.2 | 27.6 | 37 | 35.9 | 23 |
| Mexico City | Mexico | Latin America | reported | 58.8 | 2 | 38.1 | 27.2 | 10.3 | 61.6 |
| Chicago | USA | North America | reported | 66.5 | 4.6 | 25 | 57.5 | 12.5 | 26.6 |
| Los Angeles | USA | North America | reported | 66.9 | 6.3 | 25.7 | 50.9 | 23.7 | 24.4 |
| New York City | USA | North America | reported | 48.5 | 2.1 | 45 | 45 | 4.1 | 46.1 |

**Table A18: Percentage contribution to total active travel time by different main modes for older adults**

| **City** | **Country** | **Region** | **Active travel for public transport** | **Older adults (female)** | | | **Older adults (male)** | | |
| --- | --- | --- | --- | --- | --- | --- | --- | --- | --- |
|  |  |  |  | **Walk** | **Cycle** | **Public transport** | **Walk** | **Cycle** | **Public transport** |
| Accra | Ghana | Africa | harmonised | 44 | 0 | 53.7 | 71.5 | 0 | 28.5 |
| Kisumu | Kenya | Africa | harmonised | 73.4 | 2.8 | 22.5 | 85.8 | 7.8 | 6.3 |
| Cape Town | South Africa | Africa | harmonised | 50.1 | NA | 44.1 | 52.5 | 9.7 | 37.8 |
| Delhi | India | Asia | reported | 81.3 | NA | 18.7 | 83.2 | 3.6 | 13.3 |
| Melbourne | Australia | Australia | reported | 70.8 | 4.9 | 22.8 | 69.3 | 10.7 | 17.3 |
| London | England | Europe | reported | 66.3 | 1.4 | 27.6 | 62.1 | 3.9 | 28.6 |
| Berlin | Germany | Europe | harmonised | 68.2 | 10.9 | 19.3 | 63.2 | 19 | 17.7 |
| Cologne | Germany | Europe | harmonised | 74.4 | 9.8 | 13.3 | 68.7 | 20.9 | 10.4 |
| Hamburg | Germany | Europe | harmonised | 63.3 | 17.2 | 17.2 | 62.4 | 22.4 | 15.3 |
| Munich | Germany | Europe | harmonised | 54.8 | 21.4 | 22 | 54.7 | 25.5 | 19.8 |
| Zurich | Switzerland | Europe | reported | 85.6 | 4.2 | 8.4 | 85 | 7.3 | 6.2 |
| Buenos Aires | Argentina | Latin America | harmonised | 46 | 1.3 | 49.5 | 40.8 | 10.7 | 48.5 |
| Sao Paulo | Brazil | Latin America | reported | 41.9 | NA | 44.9 | 46.9 | 0.9 | 35 |
| Santiago | Chile | Latin America | harmonised | 56.6 | 0.5 | 40.5 | 45.6 | 12.5 | 41.9 |
| Bogota | Colombia | Latin America | reported | 76.9 | 0.5 | 18.9 | 63.6 | 13.1 | 19.9 |
| Mexico City | Mexico | Latin America | reported | 69.7 | 0.4 | 28.4 | 49.7 | 9.5 | 39.4 |
| Chicago | USA | North America | reported | 77.5 | 3.5 | 16.4 | 73.1 | 6.9 | 17.5 |
| Los Angeles | USA | North America | reported | 75.8 | 1.9 | 20.1 | 67 | 12 | 18.9 |
| New York City | USA | North America | reported | 53.5 | 0.3 | 40.4 | 52.8 | 1.7 | 36.5 |

**Table A19: Harmonised active travel time per capita per day (*indicates cities for which stage-level travel time was estimated using harmonisation process)**

|  |  |  |  | **All age groups combined** | | **Children** | | **Working age group** | | **Older adults** | |
| --- | --- | --- | --- | --- | --- | --- | --- | --- | --- | --- | --- |
| **City** | **Country** | **Region** | **Active travel for public transport** | **Female** | **Male** | **Female** | **Male** | **Female** | **Male** | **Female** | **Male** |
| Accra | Ghana | Africa | harmonised | 47.8 | 49.2 | 63.8 | 54.4 | 47.1 | 49.1 | 12.4 | 34.8 |
| Kisumu | Kenya | Africa | harmonised | 22.9 | 22.4 | 13.2 | 13.7 | 25.7 | 25.0 | 35.0 | 66.8 |
| Cape Town | South Africa | Africa | harmonised | 22.3 | 19.9 | 29.1 | 31.8 | 25.6 | 21.1 | 5.5 | 4.6 |
| Delhi* | India | Asia | reported | 14.1 | 21.1 | 19.4 | 18.6 | 12.0 | 23.2 | 4.9 | 11.4 |
| Melbourne* | Australia | Australia | reported | 10.5 | 10.7 | 8.8 | 8.7 | 11.7 | 11.9 | 8.2 | 9.5 |
| London* | England | Europe | reported | 23.0 | 20.5 | 14.6 | 12.8 | 27.1 | 23.9 | 19.0 | 19.3 |
| Berlin | Germany | Europe | harmonised | 34.8 | 32.2 | 31.9 | 26.1 | 35.3 | 33.0 | 35.0 | 35.1 |
| Cologne | Germany | Europe | harmonised | 34.5 | 30.3 | 34.6 | 25.8 | 37.3 | 30.9 | 28.7 | 32.6 |
| Hamburg | Germany | Europe | harmonised | 34.7 | 31.8 | 30.9 | 30.5 | 37.5 | 33.2 | 30.9 | 29.2 |
| Munich | Germany | Europe | harmonised | 36.0 | 33.4 | 36.4 | 31.1 | 36.7 | 33.4 | 34.3 | 35.5 |
| Zurich* | Switzerland | Europe | reported | 38.1 | 35.7 | 39.6 | 41.8 | 38.5 | 32.4 | 36.6 | 42.1 |
| Buenos Aires | Argentina | Latin America | harmonised | 16.9 | 14.3 | 14.4 | 14.2 | 19.9 | 15.1 | 11.1 | 11.1 |
| Sao Paulo* | Brazil | Latin America | reported | 18.8 | 18.7 | 18.4 | 18.4 | 21.2 | 20.2 | 9.4 | 11.6 |
| Santiago | Chile | Latin America | harmonised | 19.1 | 17.7 | 14.9 | 15.6 | 21.1 | 18.5 | 17.7 | 18.1 |
| Bogota* | Colombia | Latin America | reported | 24.0 | 22.9 | 19.9 | 20.0 | 26.2 | 23.6 | 21.3 | 24.9 |
| Mexico City* | Mexico | Latin America | reported | 16.5 | 14.0 | 14.0 | 14.3 | 18.2 | 14.1 | 12.4 | 12.7 |
| Chicago* | USA | North America | reported | 5.5 | 5.2 | 5.6 | 5.0 | 5.9 | 5.7 | 4.1 | 4.0 |
| Los Angeles* | USA | North America | reported | 10.5 | 10.6 | 14.1 | 13.8 | 10.8 | 10.2 | 5.6 | 8.2 |
| New York City* | USA | North America | reported | 33.3 | 32.6 | 25.8 | 23.8 | 37.0 | 36.6 | 25.6 | 30.6 |

**Table A20: Harmonised active travel time per capita per day using active travel time values from homogenous groups of the cities (*indicates cities for which stage-level travel time was estimated using harmonisation process)**

|  |  |  |  | **All age groups combined** | | **Children** | | **Working age group** | | **Older adults** | |
| --- | --- | --- | --- | --- | --- | --- | --- | --- | --- | --- | --- |
| **City** | **Country** | **Region** | **Active travel for public transport** | **Female** | **Male** | **Female** | **Male** | **Female** | **Male** | **Female** | **Male** |
| Accra | Ghana | Africa | harmonised | 47.5 | 48.5 | 63.7 | 54.1 | 46.8 | 48.2 | 12.2 | 34.0 |
| Kisumu | Kenya | Africa | harmonised | 22.9 | 22.0 | 13.2 | 13.6 | 25.7 | 24.5 | 34.8 | 66.4 |
| Cape Town | South Africa | Africa | harmonised | 22.7 | 19.6 | 29.4 | 31.4 | 26.0 | 20.7 | 5.8 | 4.5 |
| Delhi* | India | Asia | reported | 14.1 | 21.1 | 19.4 | 18.6 | 12.0 | 23.2 | 4.9 | 11.4 |
| Melbourne* | Australia | Australia | reported | 10.5 | 10.7 | 8.8 | 8.7 | 11.7 | 11.9 | 8.2 | 9.5 |
| London* | England | Europe | reported | 23.0 | 20.5 | 14.6 | 12.8 | 27.1 | 23.9 | 19.0 | 19.3 |
| Berlin | Germany | Europe | harmonised | 35.0 | 31.7 | 32.2 | 25.7 | 35.3 | 32.3 | 35.3 | 34.6 |
| Cologne | Germany | Europe | harmonised | 35.1 | 29.8 | 35.1 | 25.5 | 38.0 | 30.4 | 29.3 | 32.3 |
| Hamburg | Germany | Europe | harmonised | 35.1 | 31.3 | 31.4 | 30.2 | 38.0 | 32.5 | 31.4 | 28.9 |
| Munich | Germany | Europe | harmonised | 36.2 | 32.7 | 36.7 | 30.7 | 36.8 | 32.5 | 34.5 | 34.9 |
| Zurich* | Switzerland | Europe | reported | 38.1 | 35.7 | 39.6 | 41.8 | 38.5 | 32.4 | 36.6 | 42.1 |
| Buenos Aires | Argentina | Latin America | harmonised | 16.7 | 13.8 | 14.3 | 13.8 | 19.6 | 14.5 | 10.9 | 10.7 |
| Sao Paulo* | Brazil | Latin America | reported | 18.8 | 18.7 | 18.4 | 18.4 | 21.2 | 20.2 | 9.4 | 11.6 |
| Santiago | Chile | Latin America | harmonised | 18.8 | 16.9 | 15.0 | 15.3 | 20.7 | 17.5 | 17.5 | 17.3 |
| Bogota* | Colombia | Latin America | reported | 24.0 | 22.9 | 19.9 | 20.0 | 26.2 | 23.6 | 21.3 | 24.9 |
| Mexico City* | Mexico | Latin America | reported | 16.5 | 14.0 | 14.0 | 14.3 | 18.2 | 14.1 | 12.4 | 12.7 |
| Chicago* | USA | North America | reported | 5.5 | 5.2 | 5.6 | 5.0 | 5.9 | 5.7 | 4.1 | 4.0 |
| Los Angeles* | USA | North America | reported | 10.5 | 10.6 | 14.1 | 13.8 | 10.8 | 10.2 | 5.6 | 8.2 |
| New York City* | USA | North America | reported | 33.3 | 32.6 | 25.8 | 23.8 | 37.0 | 36.6 | 25.6 | 30.6 |

**Table A21: Percent individuals achieving at least 30 minutes of active travel time for cities that reported stage-level travel time**

| **City** | **Country** | **Region** | **All age groups combined** | | **Children** | | **Working age group** | | **Older adults** | |
| --- | --- | --- | --- | --- | --- | --- | --- | --- | --- | --- |
|  |  |  | **Female** | **Male** | **Female** | **Male** | **Female** | **Male** | **Female** | **Male** |
| Delhi | India | Asia | 24.0 | 33.0 | 32.7 | 32.9 | 20.3 | 33.9 | 13.4 | 23.0 |
| Melbourne | Australia | Australia | 15.9 | 15.7 | 12.8 | 12.1 | 17.7 | 17.5 | 13.5 | 14.1 |
| London | England | Europe | 27.4 | 25.6 | 16.8 | 15.4 | 33.0 | 30.1 | 21.5 | 23.7 |
| Zurich | Switzerland | Europe | 42.0 | 39.0 | 48.1 | 46.5 | 41.7 | 36.5 | 40.1 | 42.3 |
| Sao Paulo | Brazil | Latin America | 26.3 | 25.7 | 26.1 | 26.5 | 29.6 | 27.4 | 12.7 | 14.9 |
| Bogota | Colombia | Latin America | 27.0 | 23.5 | 25.3 | 24.1 | 28.0 | 23.1 | 25.3 | 24.6 |
| Mexico City | Mexico | Latin America | 25.6 | 21.5 | 23.1 | 23.5 | 27.7 | 21.1 | 19.8 | 19.3 |
| Chicago | USA | North America | 6.3 | 5.8 | 5.4 | 4.7 | 7.1 | 6.7 | 5.4 | 4.7 |
| Los Angeles | USA | North America | 12.1 | 11.8 | 14.5 | 15.7 | 12.4 | 11.2 | 7.5 | 8.6 |
| New York City | USA | North America | 37.1 | 35.7 | 30.4 | 29.2 | 41.5 | 39.3 | 26.6 | 29.5 |
